# Use of an Improved Matching Algorithm to Select Scaffolds for Enzyme Design Based on a Complex Active Site Model

**DOI:** 10.1371/journal.pone.0156559

**Published:** 2016-05-31

**Authors:** Xiaoqiang Huang, Jing Xue, Min Lin, Yushan Zhu

**Affiliations:** Department of Chemical Engineering, Tsinghua University, Beijing 100084, P.R. China; University of Michigan, UNITED STATES

## Abstract

Active site preorganization helps native enzymes electrostatically stabilize the transition state better than the ground state for their primary substrates and achieve significant rate enhancement. In this report, we hypothesize that a complex active site model for active site preorganization modeling should help to create preorganized active site design and afford higher starting activities towards target reactions. Our matching algorithm ProdaMatch was improved by invoking effective pruning strategies and the native active sites for ten scaffolds in a benchmark test set were reproduced. The root-mean squared deviations between the matched transition states and those in the crystal structures were < 1.0 Å for the ten scaffolds, and the repacking calculation results showed that 91% of the hydrogen bonds within the active sites are recovered, indicating that the active sites can be preorganized based on the predicted positions of transition states. The application of the complex active site model for *de novo* enzyme design was evaluated by scaffold selection using a classic catalytic triad motif for the hydrolysis of *p*-nitrophenyl acetate. Eighty scaffolds were identified from a scaffold library with 1,491 proteins and four scaffolds were native esterase. Furthermore, enzyme design for complicated substrates was investigated for the hydrolysis of cephalexin using scaffold selection based on two different catalytic motifs. Only three scaffolds were identified from the scaffold library by virtue of the classic catalytic triad-based motif. In contrast, 40 scaffolds were identified using a more flexible, but still preorganized catalytic motif, where one scaffold corresponded to the α-amino acid ester hydrolase that catalyzes the hydrolysis and synthesis of cephalexin. Thus, the complex active site modeling approach for *de novo* enzyme design with the aid of the improved ProdaMatch program is a promising approach for the creation of active sites with high catalytic efficiencies towards target reactions.

## Introduction

Designing protein active sites to catalyze chemical reactions with a designated substrate is a grand challenge of chemical biology, and the ability to design effective enzymes is ultimate proof of understanding enzyme catalysis [1]. Currently, enzymes and enzyme-based cell biocatalysts are used to develop environment-benign processes for large-scale preparation of pharmaceuticals, biofuels, and fine chemicals. Here, enzymes play key roles in eliminating bottleneck transformations during process development because of their exceptional regio- and stereoselectivity, and ambient working conditions [2, 3]. However, in most cases, enzymes isolated from natural sources are not optimal for the desired chemical transformations, or enzymes may not exist for the non-biological reactions required. To overcome the first issue, existing native enzyme scaffolds can be improved significantly by iterative rounds of directed evolution [4]. During the last two decades, directed evolution has been successfully used to strengthen enzyme stability, promote activity, or augment selectivity [5, 6]. To address the second issue, catalytic antibody technology was developed to produce catalysts with many properties of natural enzymes by mimicking the transition state (TS) of a chemical reaction [7]. Many non-biological reactions have been catalyzed by this route; however, antibody catalysts are difficult to optimize by directed evolution. A promising approach to create protein catalysts that can carry out various chemical reactions is computational enzyme design [8], which can create active sites using any type of scaffold and the low activities of the computational-designed enzymes can be amplified by directed evolution. Such advantages have helped pioneering investigators construct enzyme catalysts for several representative chemical reactions [9].

Almost seven decades ago, Pauling proposed that enzyme catalysis functions by selective stabilization of the rate-limiting TS of the catalyzed reaction relative to the ground state [10]. This realization reduces enzyme design to a structural matching problem similar to that of molecular recognition. Thus, identification of an amino acid sequence that will fold to complement the TS should bind the TS tightly and preferentially. Aided by their protein sequence selection program Orbit [11], Mayo and coworkers designed the first-generation enzyme-like proteins to catalyze the hydrolytic reaction of *p*-nitrophenyl acetate (PNPA) [12], which established the feasibility of endowing catalytic activity on an inert scaffold; although the rate enhancement was not high. Facilitated by their protein modeling program Rosetta, Baker and coworkers further developed the computational enzyme design methodology [13], where the quantum mechanically calculated theozyme model of the reaction TS is initially anchored onto the protein scaffold by the matching program RosettaMatch [14, 15]. Subsequently, residues surrounding the TS in the binding pocket are optimized to stabilize the TS by the sequence selection program RosettaDesign [16], and finally the designs are ranked and tested experimentally. RosettaMatch was experimentally confirmed to design *de novo* protein catalysts for various reactions including the Kemp elimination reaction, the retro-aldol reaction, the Diels–Alder reaction, and the ester hydrolytic reaction [17–20]. The automated searching program Dezymer was developed to construct metal-binding sites for rational design of nascent metalloenzymes [21, 22]. Other newly developed programs for matching active sites onto protein scaffolds include vector matching [23], OptGraft [24], ScaffoldSelection [25], ProdaMatch [26], AutoMatch [27], and Saber [28]. However, the actual effectiveness of these programs to generate *de novo* enzymes has not been confirmed by experimental validation [23–28].

A key merit of computational enzyme design is that the in silico designs are highly evolvable and can be optimized to high catalytic efficiencies [29]. In a recent study of the Kemp elimination reaction [30], which combined *de novo* computational design and laboratory evolution, the apparent second-order rate constant (*k*_cat_/*K*_M_) and turnover number (*k*_cat_) of the original in silico design HG3 constructed by Orbit [31] were only 430 M^−1^s^−1^ and 0.68 s^−1^, respectively [32]. After 17 rounds of directed evolution, the most active mutant HG3.17 was found to have a *k*_cat_/*K*_M_ of 2.3×10^5^ M^−1^s^−1^ and *k*_cat_ of 700 s^−1^. These values are comparable with the catalytic efficiencies of natural enzymes such as triosephosphate isomerase. However, computational design alone did not find this mutant, even though it resembles the ideal active site targeted by design. Facilitated by the crystal structure of HG3.17 with a TS analog, Blomberg *et al*. [30] found that problems in computational design may lead to low catalytic efficiency, such as ambiguous TS binding and unstable hydrogen bonding interactions between catalytic residues and TS, and can be greatly improved by laboratory evolution. The perfect hydrogen bond network formed in an active site of HG3.17 aids in the creation of a highly preorganized environment to discriminate the TS from the ground state, and electrostatic stabilization of the TS is considered the main factor of enzyme catalysis [33]. Unfortunately, current matching programs use the minimal active site model, which considers at most four catalytic residues to avoid combinatorial explosion in searching for accurate TS positions and the anchoring of suitable catalytic sites in protein scaffolds. In the active pocket of the targeted protein scaffold, there exist many hydrogen bond donor and acceptor sites from the main chain or side chains, which may compete with the TS to form hydrogen bonds with catalytic residues. Moreover, excessive degrees of freedom of the TS will lead to non-productive binding modes. Therefore, a simple minimal active site model cannot guarantee that the designed theoretical active sites will be preorganized and realized in the actual design.

In this paper, we extend the minimal active site model to include multiple residues to stabilize the TS and the key catalytic residues that interact with the TS directly. We hypothesize that the complex active site model will help to create highly preorganized active sites in actual design and afford higher starting activities to catalyze target reactions. The complex active site model consists of multiple residues and multiple constraints, which greatly increases the difficulty of finding suitable positions in protein scaffolds to anchor the TS model. ProdaMatch is a rotamer library independent matching program, which makes use of continuous optimization to handle the sampling of side chain conformations of catalytic residues and catalytic geometrical constraints. Recently, Xue *et al*. [34] developed a quasi-Newton direction-based optimization algorithm to close loops in matching. This algorithm is faster than the cyclic coordinate descent (CCD) algorithm used in the original version of ProdaMatch, and thus, larger conformational spaces can be searched rapidly. In this report, several effective pruning strategies are developed to reduce the combinatorial explosion problem for matching. The preliminary calculation results show that the improved ProdaMatch can reproduce the accurate position of the TS and the side chain conformations of active site residues based on the complex active site model for a benchmark test with ten scaffolds [15, 26]. The application of the complex active site model for *de novo* enzyme design was evaluated by scaffold selection of catalytic triad motifs for the hydrolytic reactions of PNPA and cephalexin.

## Methods

### Algorithmic improvements of ProdaMatch

A complex active site model is always composed of multiple catalytic residues and issues associated with the complicated constraints between catalytic residues and the TS for a target reaction. The model of TS and its geometrical constraints with catalytic residues can be obtained from analogy to active sites of enzymes with crystal structures, from quantum chemistry calculations [14], or from chemical intuition. This is a significant challenge for matching algorithms developed for computational enzyme design. ProdaMatch was developed to tackle this problem where the catalytic geometric parameters between catalytic residues and the TS and side chain conformations of the catalytic residues are determined by solving the loop-closure problem using a continuous optimization algorithm that avoids combinatorial explosion difficulties encountered when applying discrete optimization algorithms [26]. An overview for matching process of the original ProdaMatch algorithm is referred to Figure 2 in [26], which is also provided in this work as S1 Fig. ProdaMatch also has the advantage of being a rotamer-library-free approach. Here, the catalytic residues can adopt unusual high-energy structures that may be required by the enzyme reaction, but such geometries are not always included in conventional rotamer libraries because the rotamers in these libraries are always low-energy conformers of the naturally occurring amino acids. In the initial version of ProdaMatch [26], the loop-closure problems were solved by an extended CCD algorithm. CCD is a univariate optimization-based iterative algorithm that is inefficient because only one direction is searched in each step; yet thousands of loops need to be closed in a matching process for enzyme design and this leads to some tradeoff between matching efficiency and loop-close accuracy. Xue *et al*. [34] exemplified that inaccurate loop closure may destroy the catalytic geometric constraints between the catalytic residues and the TS. To resolve the dilemma between matching speed and loop-closure accuracy, a quasi-Newton direction based novel optimization algorithm was developed by Xue *et al*. [34] to solve the loop-closure problems in enzyme design. This algorithm has been implemented in a revised version of ProdaMatch. Compared with the CCD algorithm, the novel loop-closure algorithm is effective and efficient, and most of the initial loops for main loops or side loops can be closed under high loop-closure accuracy. This will help identify high-quality matches, but the number of closed matches increases largely and this may affect the matching efficiency of ProdaMatch for scaffold selection where hundreds of scaffolds need to be screened. Therefore, some effective pruning strategies are needed in this research based on the special characteristics of active site matching to enhance the enumeration efficiency of ProdaMatch.

The pruning strategies developed are: (i) after the candidate sites for catalytic residues are selected, the theoretical maximum lengths of a main loop or side loop can be estimated accurately before the loop closure process starts. If the maximum loop length is shorter than the true distance between the sites, the sites are pruned as unfeasible candidates. This strategy is very efficient in trimming unnecessary loops and can reduce the combinatorial number greatly. However, maximization of a loop length is a complicated global optimization problem that depends on many variables, including bond lengths, bond angles, and torsion angles within a loop. Here, we introduce a simple but highly efficient approach to calculate the theoretical maximum length of a loop. After two sites are specified to anchor a loop, the starting CA atom and terminal CA atom of this loop are determined. To facilitate the calculation, a dummy CA atom is generated and located along the direction of the vector from the starting CA atom to the terminal CA atom with a distance of 1,000 Å starting from the starting CA atom. The loop closure process is run to minimize the distance between the calculated terminal CA atom and the dummy CA atom. This loop closure process always converges after a few iteration steps, and the distance between the starting CA atom and the calculated terminal CA atom is considered as the maximum length of the incumbent loop. (ii) The loop-closure problem is always initial-value dependent. This indicates that some of the initial loops of a main loop or side loop may finally converge to the same geometries; although such initial loops are different from each other from at least a torsion angle standpoint. Therefore, the converged loops can be eliminated if their root mean standard deviations (RMSDs) with the stored loops are smaller than a predetermined threshold. (iii) If the catalytic geometric constraints are given between the atoms from the incumbent loop and atoms from backbone or stored loops, these constraints can be checked immediately after the incumbent loop is closed. If any constraint does not hold, the incumbent loop is discarded. (iv) After the loop closure processes are finished for main loops or side loops, the van der Waals repulsive energies between the closed loops and the backbone or template are calculated according to a linear repulsive energy term that is described by Equation 2 in our previous work [35]. Here, backbone refers to the main chain atoms of the residues at the candidate sites, while the template refers to atoms of all residues not at the candidate sites. If the energy scores are greater than the predetermined thresholds, the closed loops are discarded. The threshold energy value is set at 150 Proda energy units (PEU) for the energy between the closed main loop and the backbone, 30 PEU between the closed side loop and the backbone, 200 PEU between the whole match and the template, and 50 PEU between the whole match and the template. These values are assigned based on the calculated energies for the native matches in a benchmark set complied by Zanghellini *et al*. [15].

### Optimization of side chain conformations in active sites

The accurate position of the TS is critical for enzyme catalysis. To evaluate the effect of the TS position on catalytic efficiency, side chain conformations of residues surrounding the TS state are optimized by the repacking calculation while the position of the TS is constrained to that determined by the revised matching algorithm ProdaMatch, which is based on either the conventional minimal active site model or the complex active site model. The side chain repacking calculation is implemented by the systematic optimization model and algorithm for enzyme design, as reported previously [36]. For any given scaffold, residues that lie within 7.0 Å of the TS in the crystal structure are selected as design sites for side chain conformational optimization, except for prolines. The side chain conformations of each amino acid are taken from the backbone independent rotamer library complied by Xiang and Honig [37]; the number of rotamers in this original library is 11,810. The rotamers for serine, threonine, and tyrosine are expanded (see [35]) to compute the hydrogen-bonding interaction accurately because the hydroxyl hydrogen atom of these residues can adopt multiple positions. The atomic parameters and internal coordination parameters for amino acids are taken from the all-atom force field CHARMM 22 [38], which considers hydrogen atoms explicitly. The atomic parameters for TS are constructed by similar atoms from the model compounds of CHARMM 22 [38] and atomic partial charges for TS are assigned based on either the CHARMM 22 charges [38], or PARSE charges [39]. The free energy of the protein-ligand system is calculated by the energy function developed in our earlier work [35, 36], which is based on molecular mechanics and the continuum solvent model. Side chain conformation optimization is solved by the combined Dead End Elimination/Linear Programming/Mixed-Integer Linear Programming (DEE/LP/MILP) algorithm [35, 36].

### Scaffold library construction and scaffold selection

A scaffold library is constructed in three steps to provide input files for active site matching. First, a set of 8,296 protein entries is selected from the Protein Data Bank (PDB) that satisfies the following conditions as: (i) The protein has been expressed previously in *Escherichia coli*; (ii) The protein structure has been determined by X-ray crystallography and the structural resolution is < 3.0 Å; (iii) The number of residues of the protein lies between 300 and 800; (iv) The sequence identity between any two proteins in this set is lower than 95%. Second, a set of 25,358 protein entries is obtained from the enzyme structure database, Catalytic Site Atlas (CSA) [40]. Third, an intersection set of the above two sets is generated, which contains 1,491 protein entries and their structure files are downloaded directly from the PDB and saved as the scaffold library for active site matching. All proteins in the scaffold library are enzymes and only enzymes were chosen as scaffolds because multiple mutations are always introduced for *de novo* computational enzyme design and the active site pocket of an enzyme has evolved to tolerate multiple mutations. The protein entries in the scaffold library are listed in S1 Table.

The scaffold selection is carried out by running the active site matching algorithm ProdaMatch on each scaffold in the library. The candidate sites for matching on each scaffold are selected based on the sites of the native catalytic residues of that scaffold, which are recorded in the CSA file. The centroid coordinates of the CA atoms of the native catalytic residues are calculated for each scaffold, and the residues that lie within 15 Å to the centroid are selected as candidate sites for matching. Several matches may be obtained on one scaffold and the position of the TS in each match is checked by visual inspection. If the TS is located outside of the pocket of the scaffold, the match is discarded. If no feasible match is identified by ProdaMatch, the scaffold is discarded. The scaffolds selected by ProdaMatch with feasible matches for the target active site model are subjected to further design. All calculations are performed on a computer cluster with 256 cores, where each core represents a single 2.1-GHz central processing unit (CPU) from a sub-cluster with 64 cores that shared 128 GB of random-access memory. ProdaMatch program is available by sending request to the corresponding author of this work.

## Results

### Recapitulation of native active sites using revised ProdaMatch based on the complex active site model

**Fig 1:**
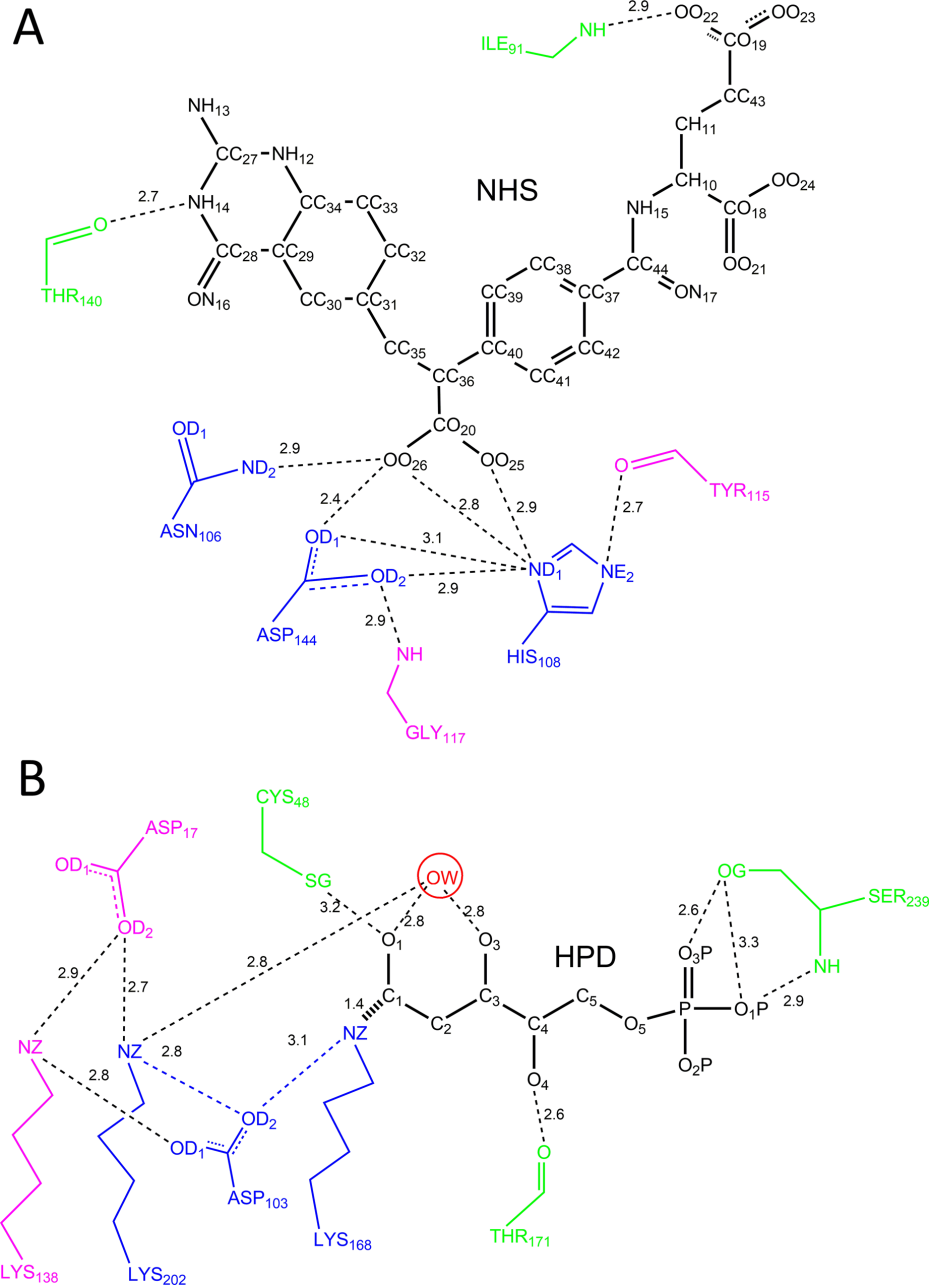
**Schematic diagram of complex active site models for scaffolds: (A) 1c2t; (B) 1jcl.** The catalytic residues are colored in blue, the residues that stabilize the catalytic residues are colored in pink, and the residues that stabilize the TS are colored in green.

The complex active site model proposed in this research is composed of a set of residues including: (i) the catalytic residues; (ii) the residues that stabilize the catalytic residues; and (iii) the binding residues that stabilize the TS. To circumvent combinatorial explosion during the matching process for site selection in scaffolds, the set of residues in a complex active site model was always minimized. Consistent with the definition of catalytic residues proposed by Thornton and colleagues [41], the active site residues were classified as catalytic because they are directly involved in the catalytic mechanism, such as the nucleophile, aiding the catalysis by electrostatic or acid-base action, or stabilizing the proposed TS intermediate. The residues for stabilizing the catalytic residues are included in the complex active site model because they help to position the catalytic residues accurately to maintain the catalytic geometric relationships between the catalytic residues and the TS. Only the binding residues that play pivotal roles in anchoring the TS are selected to participate in the complex active site model. The benchmark test set of ten enzyme-TS or enzyme-inhibitor complexes compiled by Zanghellini *et al*. [15] was used to test the improved ProdaMatch algorithm, which is shown in Table 1. Illustration of the complex active site model and its counterpart the minimal active site model for scaffolds 1c2t and 1jcl is shown in Fig 1, and the other eight scaffolds are shown in S2–S9 Figs. The catalytic constraint parameters for matching the ten examples are described in S2–S21 Tables for both active site models, where the optimal values for distances, angles, and improper dihedral angles are taken directly from the crystal structures.

**Table 1:** Recapitulation of native active sites by ProdaMatch for ten scaffolds.

PDB	Active site residues ^a^	No.sites ^b^	Rank/No.matches ^c^	RMSD of TS ^d^
1c2t	Asn106, His108, Asp144 / Gly117, Tyr115 / Thr140, Ile91	44 (27)	1/1 (20/28)	0.24 (7.56)
1dqx	Asp91, Asp273, Lys93, Lys59 / Ser35 / Ser154, Gly234, Thr277	29 (29)	1/2 (37/16164)	0.28 (1.79)
1h2j	Glu225, Glu136 / His197, Tyr199 / Tyr63, Ala231, Trp259	24 (24)	1/19 (39/190)	0.42 (3.94)
1jcl	Lys168, Asp103, Lys202 / Asp17, Lys138 / Cys48, Thr171, Ser239	33 (23)	1/4 (56/709)	0.14 (3.77)
1ney	Glu164, His94, Lys11 / Glu96 / Gly231, Gly170	20 (20)	1/5 (2/222)	0.65 (1.73)
1oex	Asp35, Asp217 / Ser38, Thr220 / Gly219, Gly78	21 (21)	1/2 (4/35)	0.28 (6.31)
1p6o	Cys89, Cys92, His60, Glu62 / / Asn49, Asp153	18 (18)	1/2 (10/28)	0.66 (1.25)
3vgc	Ser175, His42, Asp87 / Ser194 / Gly173	34 (34)	1/2 (7/26)	0.55 (1.75)
4fua	His155, His94, His92 / / Thr43, Gly44	19 (19)	1/133 (1/83)	0.33 (2.96)
6cpa	His69, Glu270, His196, Glu72 / Asp142 / Arg127	23 (23)	1/4 (1/156)	0.88 (1.47)

^a^: Active site residues are classified into three groups and separated by slash. The first group represents catalytic residues, the middle group represents the residues which stabilize the catalytic residues, and the last group represents the residues which stabilize the TS. The residues in the minimal active site model correspond to those in the first group.

^b^: The column “No. sites” shows the number of candidate sites for complex active site model, and the numbers in the parentheses are for the minimal active site model.

^c^: The column “Rank/No. matches” shows the rank of the native match among all identified matches based on complex active site model, and the numbers shown in the parentheses are for minimal active site model.

^d^: The column “RMSD of TS” shows the RMS deviations of the matched TS based on the complex active site model from that in the crystal structures, and the values shown in the parentheses are for minimal active site model.

**Fig 2:**
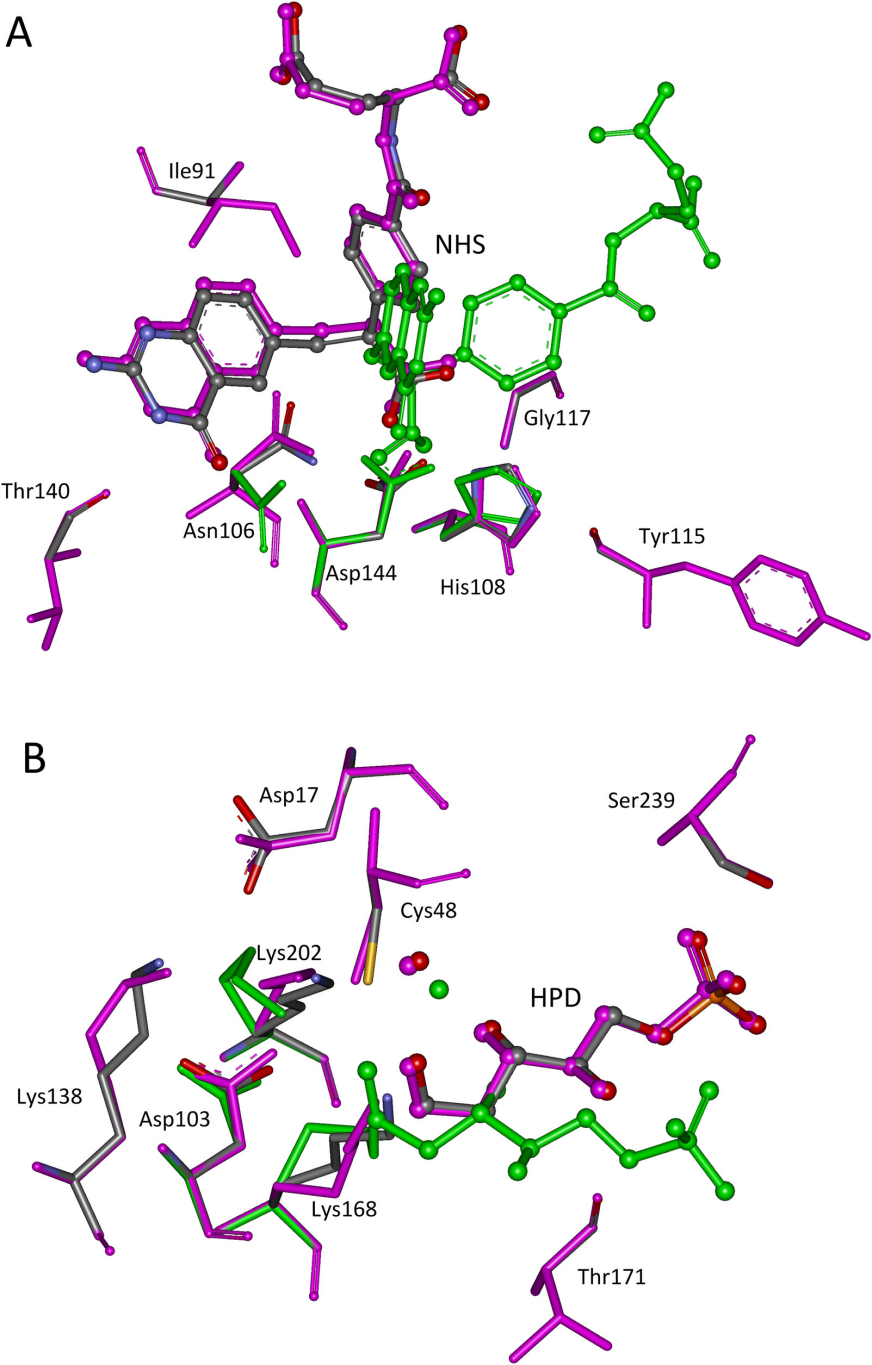
Superposition of native and predicted active sites. **(A) 1c2t; (B) 1jcl.** The transition states are shown in ball and stick model, and the active site residues in stick model. Atoms O, N, and C in crystal structures are colored in red, teal, and gray, respectively. The matched structures based on complex active site model are shown in pink, and in green for matched structures based on minimal active site model.

The native active site recapitulation results obtained by the revised ProdaMatch for ten scaffolds in the benchmark set are given in Table 1. All native matches for ten scaffolds are identified by the revised ProdaMatch and ranked first among all matches based on the complex active site model. For the conventional minimal active site model, native matches rank first for only two scaffolds. Nonetheless, the conventional minimal active site model does identify native matches for the ten scaffolds. Moreover, the number of matches obtained by virtue of the complex active site model for all scaffolds, except 4fua, is much less than that by virtue of the minimal active site model. These results imply that the revised ProdaMatch can discriminate between the native matches and the non-native matches effectively based on the complex active-model, and can be attributed to the introduced catalytic geometric constraints in the complex active site model, which helps to reduce the total number of matches and eliminate unnecessary non-native matches. The CPU running time for matching is given in S22 Table for the ten scaffolds and it shows that combinatorial explosion was never encountered by the revised ProdaMatch even though the number of active site residues reached eight for some scaffolds, such as 1dqx and 1jcl. Furthermore, the CPU time is feasible for scaffold selection when parallel computing is used. It should be noted that not only are the native active sites recaptured for the ten scaffolds, but also the TS positions and side chain conformations of the active site residues in the identified native matches are reproduced accurately based on the complex active site model. The RMSDs of the matched TS for ten scaffolds from those in the crystal structures are less than 1.0 Å, as shown in Table 1, which is much better than the results obtained by the minimal active site model. The matched active site structures for 1c2t and 1jcl based on the complex and minimal active site models as well as the crystal structures are shown in Fig 2, and the results for other scaffolds are given in S10–S17 Figs. The RMSDs of the active site residues for ten scaffolds from those in the crystal structures are shown in S22 Table. The RMSDs presented in S22 Table show that the side chain conformations of the active site residues are better reproduced based on the complex active site model than those based on the minimal active site model.

To investigate the effect of the matched TS position on enzyme catalysis, the side chain conformations of the residues surrounding the TS are optimized with the TS fixed at the matched position. The binding energy between an enzyme and TS determines the catalytic efficiency of the enzyme, and the binding energy is calculated as the energy difference between the bound enzyme-TS system and the unbound enzyme-TS system. Three scaffolds, 1p6o, 4fua, and 6cpa, in the benchmark set are not considered for repacking calculations because these three scaffolds contain metal ions in their active site pockets and the energy function in PRODA cannot model metal coordination. Hydrogen-bonding interactions are abundant in biological systems, and they always play critical roles in molecular recognition between protein and ligand. The recovered hydrogen bond numbers and the calculated binding energies for the seven scaffolds in the benchmark test set are given in Table 2. Detailed information about which hydrogen bonds are recovered and which are not is further shown in S23 Table.

**Table 2:** Side chain repacking results for seven scaffolds.

PDB	Hydrogen bonds			Binding energy (Proda energy units)		
	Native	Minimal	Complex	Native	Minimal	Complex
1c2t	13	5 (38%)	13 (100%)	-68.48	38.07	-69.30
1dqx	19	3 (16%)	17 (89%)	-52.35	30.67	-54.10
1h2j	13	3 (23%)	10 (77%)	-37.72	144.61	-40.15
1jcl	11	4 (36%)	11 (100%)	-30.33	35.20	-28.01
1ney	14	8 (57%)	11 (79%)	-42.98	-18.93	-39.10
1oex	9	6 (67%)	9 (100%)	-57.80	-18.28	-53.86
3vgc	9	8 (89%)	9 (100%)	-30.31	31.77	-30.45
Total	88	37 (42%)	80 (91%)	-	-	-

The columns “Native”, “Minimal”, and “Complex” shows results calculated by TS in the crystal structures, matched TS based on the complex active site model and the minimal active site model, respectively.

**Fig 3:**
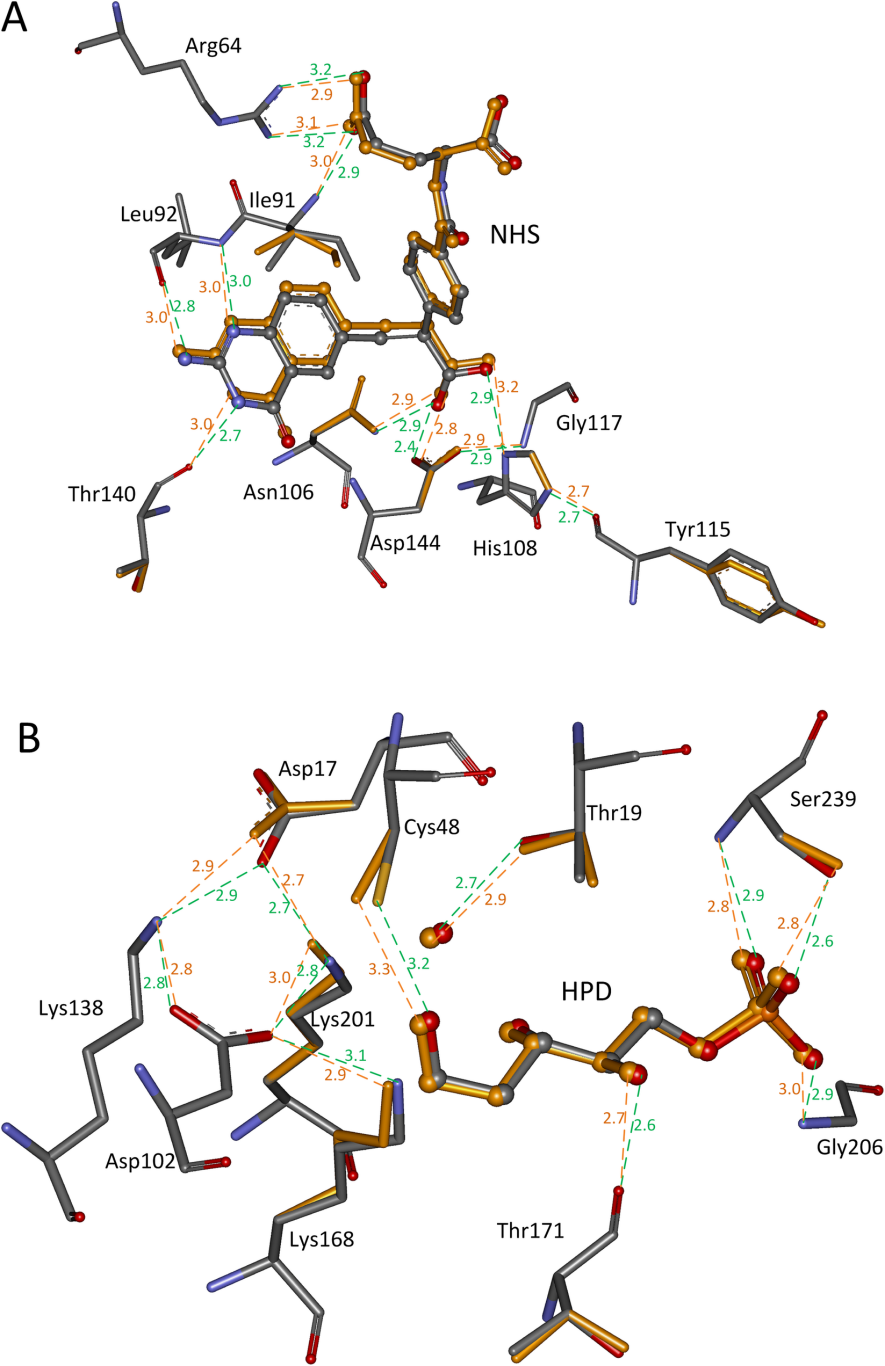
**Side chain repacking results for scaffolds: (A) 1c2t; (B) 1jcl.** The transition states are shown in ball and stick model, and the active site residues in stick model. Atoms O, N, and C in crystal structures are colored in red, teal, and gray, respectively. The matched TS and repacked residues are colored in orange. The hydrogen bonds in crystal structures are shown in dotted green lines, and the predicted hydrogen bonds are shown in dotted orange lines. The distances between hydrogen bonding donors and acceptors are shown in Å and labeled besides the dotted lines.

When the TS position is determined by the complex active site model, 91% (80/88) of the hydrogen bonds in the active sites of the seven scaffolds are recovered, and all hydrogen bonds in four scaffolds, i.e., 1c2t, 1jcl, 1oex, and 3vgc have been recovered. In contrast, only 42% (37/88) of the hydrogen bonds were recovered when the TS position is determined by the minimal active site model. The repacked active site structures for scaffolds 1c2t and 1jcl are shown in Fig 3, and the geometries of the matched TS and repacked side chains of the surrounding residues overlap perfectly with those in the crystal structures. The calculated binding energies for seven scaffolds shown in Table 2 are consistent with those in the crystal structures when the TS position is determined by the complex active site model. When the TS position is inaccurate, which is determined by the minimal active site model, the calculated binding energies are always not plausible because some binding energies are positive. The amino acid sequence in the active site of an enzyme is always determined by the binding energy; therefore, an accurate TS position is critical for computational enzyme design.

### Scaffold selection of a hydrolytic reaction based on the classic catalytic triad motif

**Fig 4:**
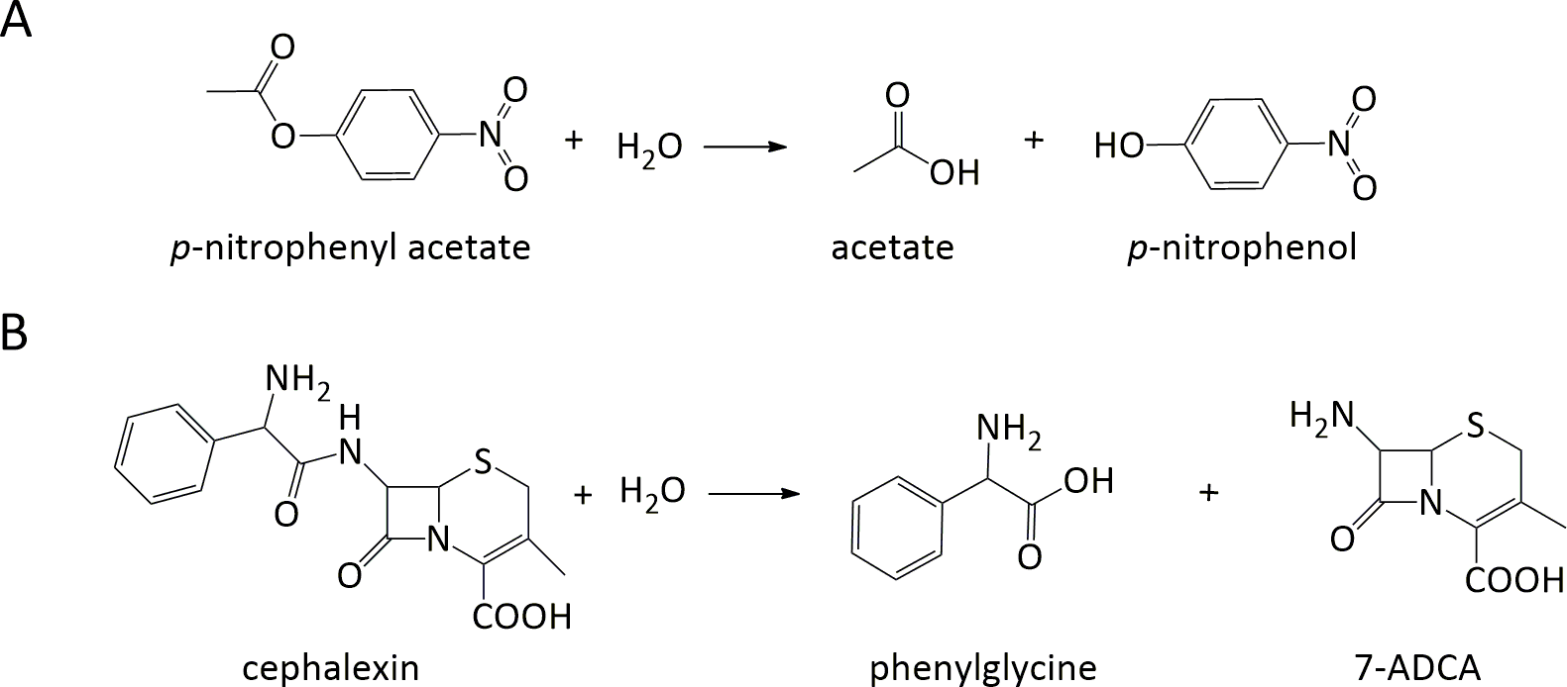
Reaction schemes of hydrolysis of PNPA and cephalexin. PNPA: *p*-nitrophenyl acetate; 7-ADCA: 7-amino desacetoxycephalosporanic acid.

**Fig 5:**
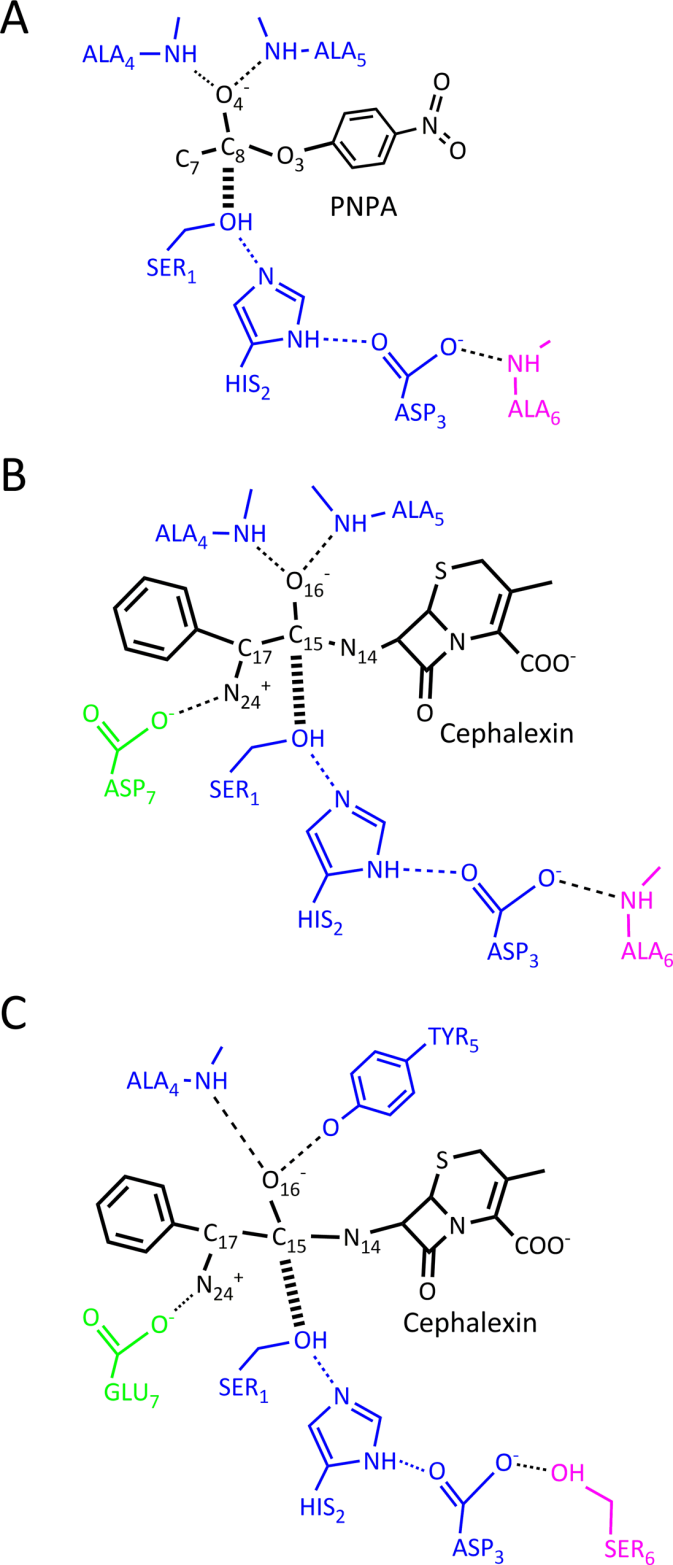
Complex active site models for PNPA and cephalexin based on different catalytic motifs. (A) Classic catalytic triad motif for hydrolysis of PNPA; (B) Classic catalytic triad motif for hydrolysis of cephalexin; (C) Flexible catalytic triad motif for hydrolysis of cephalexin.

The revised ProdaMatch has been confirmed by the benchmark test set to anchor the native active sites on native scaffolds perfectly when using the complex active site model, but its application for *de novo* enzyme design needs to be examined by identifying suitable scaffolds from a scaffold library for a target reaction. The target reaction chosen here is the hydrolytic reaction of PNPA and the reaction scheme is shown in Fig 4A. This reaction is the first case study for computational enzyme design [12], where one histidine is used as the nucleophile and the *de novo* design of a protein catalyst for the hydrolytic reaction using the classic triad mechanism has never been realized [20, 42]. The complex active site model for the hydrolytic reaction of PNPA based on the tetrahedral TS is shown in Fig 5A, where the classic catalytic triad Ser-His-Asp is used as the nucleophile, the negatively charged oxyanion is stabilized by two backbone hydrogen bonds and an additional backbone hydrogen bond is used to stabilize the aspartate. The catalytic geometric parameters for this active site model are presented in S24 Table, which were obtained from the crystal structures of the hydrolases [43] and used as the unified matching parameters for scaffold selection.

The scaffolds were searched by the revised ProdaMatch in the constructed scaffold library with 1,491 protein entries, and 80 scaffolds were identified that could afford the active site of the hydrolytic reaction of PNPA. These scaffolds include members of oxidoreductase, transferase, hydrolase, lyase, isomerase, and ligase enzyme families. The 80 scaffolds and the number of their respective matches are listed in Table 3.

**Table 3:** Selected scaffolds by ProdaMatch for hydrolytic reactions of PNPA and cephalexin using catalytic triad based motifs.

Substrates	Active site residues	Selected scaffolds (Number of matches)
PNPA	SER1, HIS2, ASP3, ALA4, ALA5, ALA6	12as(1), 1a59(1), 1azw*(11), 1b6g(1), 1czi(3), 1f6w*(6), 1fl2(3), 1g6s(1), 1ii2(1), 1iyk(1), 1jkm*(12), 1l5w(1), 1l8k(7), 1l9x(1), 1lf2(1), 1o7x(1), 1odt(2), 1pj3(2), 1q7e(1), 1qe3* (9), 1qh4(1), 1qo5(1), 1tzj(1), 1u6r(2), 1vdc(3), 1w6t(1), 1wm1* (7), 1wos(1), 1xql(4), 1zai(3), 1zod(1), 2a9d(2), 2aeb(1), 2bht(2), 2c7s(5), 2cfv(1), 2cjz(4), 2cm2(5), 2cxe(1), 2d1f(1), 2dbq(1), 2dfd(1), 2dw4(1), 2dyu(1), 2es4* (9), 2f00(1), 2h4v(2), 2hsa(2), 2i6u(1), 2iwz(1), 2ix4(2), 2j07(2), 2jbv(1), 2nlk(3), 2o7r* (6), 2q3m(1), 2q4w(2), 2qjf(1), 2r11* (6), 2vba(1), 2xgz(3), 2zsi(5), 3b7o(6), 3emc(1), 3exe(2), 3hjb(1), 3ho9(1), 3ju5(2), 3lwb(1), 3m83* (7), 3mbd(1), 3nvs(1), 3ru6(1), 3sk0(1), 3tlo(1), 3tqp(1), 3vmf(2), 3zwc(1), 4amv(1), 4j1y(3)
Cephalexin	SER1, HIS2, ASP3, ALA4, ALA5, ALA6, ASP7	1azw* (2), 1wm1*(2), 3m83*(3)
Cephalexin	SER1, HIS2, ASP3, ALA4, TYR5, SER6, GLU7	1b1y(2), 1ei5(2), 1g0d(3), 1gsa(1), 1hso(3), 1ji1(3), 1jkm*(17), 1jvb(17), 1mla(208), 1mpx*(35), 1owl(4), 1qak(11), 1qj5(24), 1tel(1), 1u3u(4), 1u3w(1), 1uqt(3), 1v8b(3), 1wdk(13), 2dpl(18), 2f00(17), 2fpq(5), 2hg2(1), 2ode(6), 2wzb(2), 2xdw(53), 2z3z(37), 3d2f(1), 3fpc(3), 3ho9(3), 3i2k*(476), 3iar(55), 3im9(22), 3mvi(155), 3ond(1), 3prh(2), 3tqp(5), 3tx1(2), 3uko(17), 4edf(11)

*The asterisks following the PDB codes indicate that the scaffolds have native catalytic triads in their active pockets.

**Fig 6:**
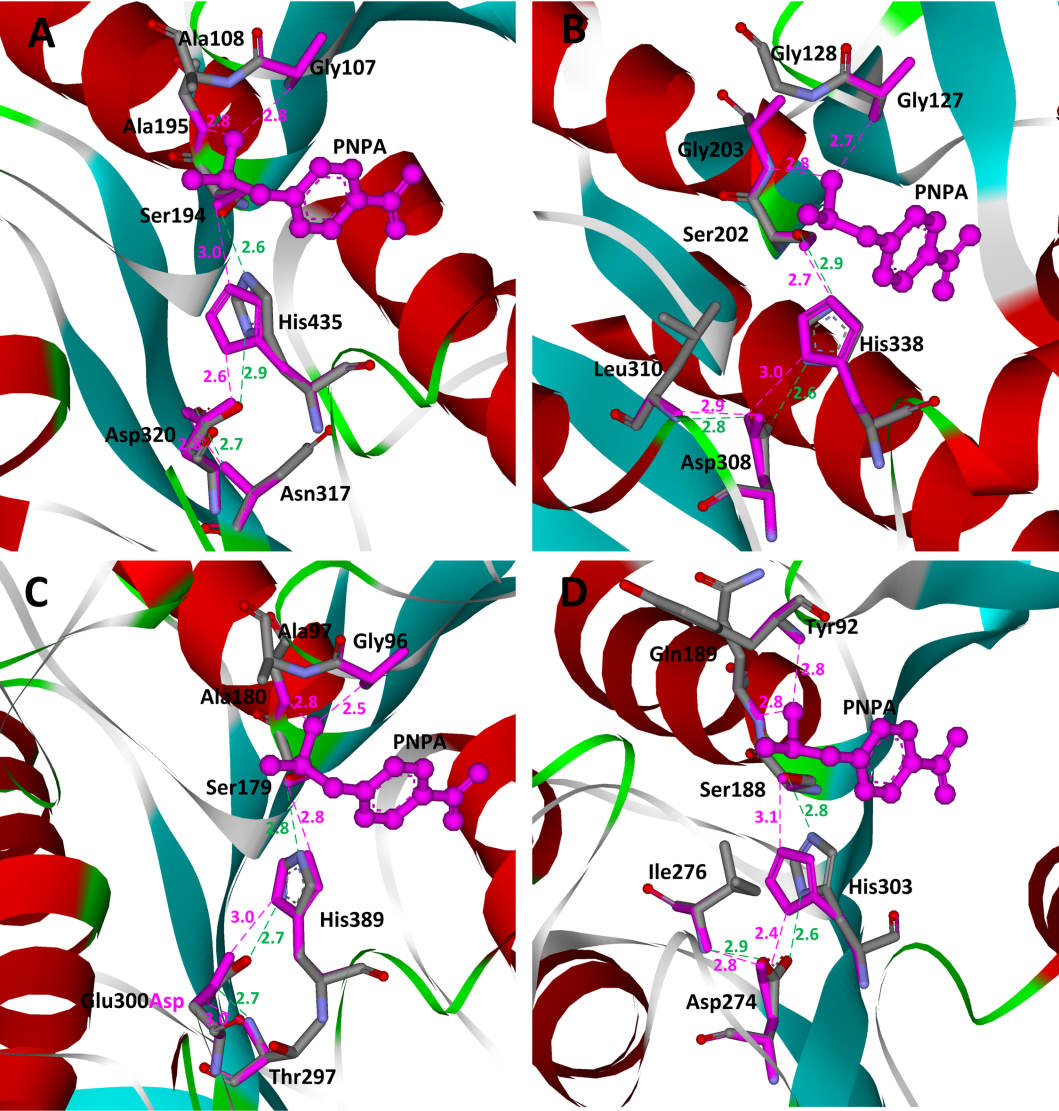
Superposition of native and predicted active sites for hydrolysis of PNPA on scaffolds. **(A) 1f6w; (B) 1jkm; (C) 1qe3; (D) 3m83.** The transition states are shown in ball and stick model and colored in pink. The active site residues are shown in stick model. Atoms O, N, and C in crystal structures are colored in red, teal, and gray, respectively. Matched residues are colored in red. The hydrogen bonds in crystal structures are shown in dotted green lines, and the predicted hydrogen bonds are shown in dotted pink lines. The distances between hydrogen bonding donors and acceptors are shown in Å and labeled besides the dotted lines.

Among the 80 scaffolds, four scaffolds, i.e., 1f6w, 1jkm, 1qe3, and 3m83, are native enzymes that catalyze the hydrolysis of PNPA and are identified by ProdaMatch successfully. The matched active site residues and their crystal structures as well as the TS for these four scaffolds are shown in Fig 6. All native active sites are found to be perfectly reproduced. Specifically, 1f6w is the crystal structure of the catalytic domain of human bile salt-activated lipase [44], and six matches were identified on1f6w. In all six matches, the catalytic triad residues are anchored at Ser194, His435, and Asp320, and the Asp stabilizing residue is anchored at Asn317. The oxyanion stabilizing residues are anchored at Ala195, Gly107, or Ala108, and by virtue of the crystal structure shown in Fig 6A, the oxyanion, i.e., atom O4 of PNPA, can form perfect hydrogen bonds with the amide groups of these three residues. Therefore, all six matches identified for scaffold 1f6w are native matches for the hydrolytic reaction of PNPA, and the top ranked match sites at Ala195 and Gly107 to anchor the oxyanion-stabilizing residues. 1jkm is the crystal structure of brefeldin A esterase [45] from *Bacillus subtilis*. In the crystal structure of 1jkm shown in Fig 6B, the native catalytic triad residues are Ser202, His338, and Asp308, respectively. Three oxyanion stabilizing residues are Gly203, Gly127, and Gly128, and the additional aspartate-stabilizing residue is Leu310. Twelve matches were identified on 1jkm, where four of them are native matches. The highest native match was ranked third, but the geometries between the catalytic triad of the top two matches are very poor because the hydrogen bonding angles deviate largely from optimal values. 1qe3 is the crystal structure of *p*-nitrobenzyl esterase [46] from *Bacillus subtilis*. In the crystal structure of 1qe3 shown in Fig 6C, the native catalytic triad residues are Ser189, His399, and Glu310. The three oxyanion stabilizing residues are Ala190, Ala107, and Gly106, and the additional aspartate-stabilizing residue is Thr307. Nine matches were identified in 1qe3 and five of them have anchored the active site model residues at the corresponding native sites; only the native residue Glu310 was mutated to Asp310 because Asp is used to comprise the catalytic triad in our active site model for matching. By virtue of the critical structure shown in Fig 6C, residue Ser215 forms an additional hydrogen bond with the carboxylate of Glu310. The side chain of glutamate is more flexible than that of aspartate, and this explains why two hydrogen bonds are used to stabilize the Glu310 in the native enzyme. 3m83 is the crystal structure of a thermostable acetyl esterase [47] from *Thermotoga maritima*. In the crystal structure of 3m83 shown in Fig 6D, the native catalytic triad residues are Ser188, His303, and Asp274. The two oxyanion stabilizing residues are Gln189 and Tyr92, and the two additional aspartate stabilizing residues are Ile276 and Cys277. Seven matches were identified in 3m83 and two of them are native matches. In the native structure of 3m83, two residues, Ile276 and Cys277, form hydrogen bonds with the catalytic triad Asp, but in our active site model only one residue is used to stabilize Asp. This probably explains why the two native matches are not ranked first.

### Effect of different catalytic motifs on scaffold selection

In the catalytic motif for the hydrolytic reaction of PNPA shown in Fig 5A, two backbone hydrogen bond donors are used to stabilize the oxyanion and one to stabilize Asp in the catalytic triad. The backbone hydrogen bonds are always fixed in any scaffolds and this helps in the preorganization of the matched active sites. However, the backbone hydrogen bonds hardly have any conformational degrees of freedom and this greatly restricts the selection of scaffolds if the substrate is large and has a complex structure. To investigate the effect of the catalytic motif on active site design, we have carried out scaffold selection for the hydrolytic reaction of a semi-synthesized antibiotic cephalexin by considering different catalytic motifs. The reaction scheme is shown in Fig 4B and the hydrolysis of cephalexin is the reverse of its synthesis. Thus, this reaction is of crucial significance in pharmaceutical preparation. Because an amide bond is much less reactive than an ester bond [48], the catalytic triad functions to break the amide bond. The complex active site model for hydrolysis of cephalexin is constructed using the same catalytic motif used for the hydrolysis of PNPA, which is shown in Fig 5B; however, an extra aspartate is added to stabilize the positively charged α-amino group of cephalexin. The catalytic geometric parameters for this active site model are presented in S25 Table and used as the unified matching parameters for scaffold selection. The scaffolds were searched from the scaffold library with only three scaffolds identified that could afford the active site of the hydrolysis of cephalexin. The list of the three scaffolds, i.e., 1azw, 1wm1, and 3m83, and the number of their respective matches are shown in Table 3. Compared with the scaffold selection results for the hydrolysis of PNPA, the identified scaffolds and the total number of matches for the hydrolysis of cephalexin are greatly reduced. A literature survey shows that the native enzymes of the three identified scaffolds are not found to have any catalytic activity towards the hydrolysis of cephalexin, although scaffolds 1azw and 1wm1 have native catalytic triads. The size of cephalexin is much larger than that of PNPA, and there are two large ring groups in cephalexin that are located on either side of the amide bond. These structural complexities are not compatible with the rigidity of the catalytic motif shown in Fig 5B; therefore, the number of selected scaffolds is greatly reduced. Consequently, a different catalytic motif with greater conformational flexibility needs to be constructed for the hydrolysis of cephalexin.

**Fig 7:**
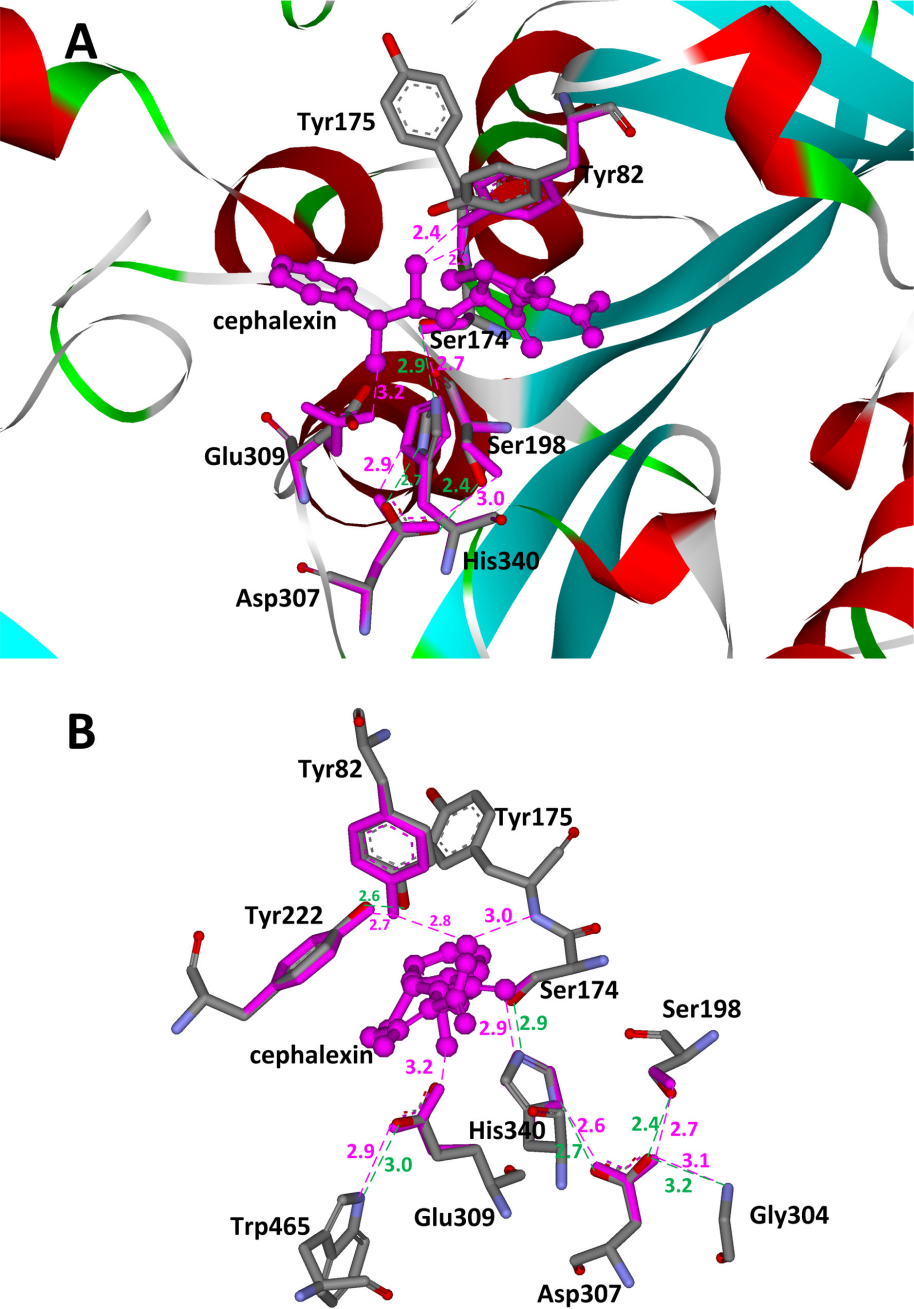
Matching results and active site residue sidechain repacking results in scaffold 1mpx. (A) Superposition of native and matched active sites for hydrolysis of cephalexin on scaffold 1mpx. (B) Conformations of repacked residues based on matched cephalexin on scaffold 1mpx. The transition states are shown in ball and stick model and colored in pink. The active site residues are shown in stick model. Atoms O, N, and C in crystal structures are colored in red, teal, and gray, respectively. Matched residues are colored in red. The hydrogen bonds in crystal structures are shown in dotted green lines, and the predicted hydrogen bonds are shown in dotted pink lines. The distances between hydrogen bonding donors and acceptors are shown in Å and labeled besides the dotted lines.

We observe that the side chains of particular amino acids in many native serine hydrolases are also used to donate hydrogen bonds to stabilize the oxyanion besides the backbone. For example, the side chain amide of an asparagine forms a hydrogen bond with the oxyanion in penicillin G acylase and glutaryl acylase [49–51], and the side chain hydroxyl of tyrosine plays the same role in the α-amino acid ester hydrolase from *Xanthomonas citri* and the bacterial cocaine esterase [52, 53]. In the preceding section, we observed that serine is used to stabilize the catalytic triad aspartate or glutamate in particular scaffolds, such as 1f6w, 1mpx, and 1qe3. Therefore, we have modified the catalytic motif shown in Fig 5B for the hydrolysis of cephalexin. Here, one backbone hydrogen bond donor in the oxyanion hole is substituted by the side chain of a tyrosine, the side chain hydroxyl group of a serine is used to stabilize the catalytic triad aspartate, and a glutamate rather than the aspartate is used to stabilize the α-amino group of cephalexin. The complex active site model based on the above catalytic motif is shown in Fig 5C, and the catalytic geometric parameters for this model are presented in S26 Table and used for scaffold selection. Forty scaffolds were identified that could afford the complex active site of the hydrolysis of cephalexin. These scaffolds include members of oxidoreductase, transferase, hydrolase, lyase, isomerase, and ligase enzyme families. The list of these 40 scaffolds and the number of their respective matches are shown in Table 3. Among all identified scaffolds, one scaffold, i.e., 1mpx, is the structure of the α-amino acid ester hydrolase [52] from *X*. *citri*, which catalyzes the hydrolysis and synthesis of some β-lactam antibiotics such as cephalexin, cefatrizine, ampicillin, and amoxicillin. In scaffold 1mpx, the native catalytic triad residues are Ser174, His340, and Asp307. The oxyanion hole comprises the backbone amide group of Tyr175 and the side chain amide group of Tyr82. The hydroxyl group of Ser198 forms a hydrogen bond with the catalytic triad Asp307 and the carboxyl group of Glu309 is employed to stabilize the α-amino group of cephalexin. Thirty-five matches were identified by the revised ProdaMatch in this scaffold and the native match ranks tenth among all matches. In all matches, the catalytic triad nucleophile Ser174 and the backbone oxyanion hole Tyr175 are identified correctly. The matched native active site and the crystal structure are compared in Fig 7A. It can be seen that all the matched residues choose similar conformations compared with that of the native structure. Actually, by virtue of the crystal structure of scaffold 1mpx, we found that the hydroxyl group of the oxyanion hole residue Tyr82 is stabilized by the hydroxyl group of Tyr222, and there is a hydrogen bond formed between the carboxyl group of Glu309 and atom NE1 of Trp465 that is tightly packed in the active site. The side chains of residues that lie within 7.0 Å of the matched TS of cephalexin were repacked without considering the catalytic constraints between active site residues and the calculation results are shown in Fig 7B. This figure shows that all hydrogen bonds depicted in the complex active site model and the two additional hydrogen bonds between Tyr82 and Tyr 222, and Glu309 and Trp465 are constructed with perfect geometries. These observations imply that the native enzymes have employed complex catalytic motifs to realize the preorganization of their active sites for complexreactions.

## Discussion

In this work, we proposed the concept of a complex active site model to capture the major defining characteristics of the active site preorganization towards a target reaction, and the native active sites in a benchmark set were reproduced accurately based on this model with aid of an improved version of our matching algorithm ProdaMatch for enzyme design. The complex active site model was further validated by scaffold selection from a library with 1,491 protein entries based on classic and flexible catalytic triad motifs for the hydrolysis of two standard chemical compounds. Active site matching programs RosettaMatch [15], vector matching [23], ScaffoldSelection [25], AutoMatch [27] and Saber [28] have been tested for *in silico* enzyme design on different scaffold databases. Similar to these programs, ProdaMatch could identify all native enzymes and rank native matches high for model chemical reactions.

Accurate position of the TS is critical for enzyme catalysis. However, manipulation of the TS position in enzyme design is not trivial as the translational, rotational, and conformational degrees of freedom of the TS are not easily determined accurately by computational design. This was demonstrated by the recent enzyme design for the Kemp elimination reaction [30, 32], where the ligand had undergone a large change in its pose in the most active mutant HG3.17 obtained by 17 rounds of laboratory evolution from its pose in the original computational design HG3 developed by Orbit and molecular dynamics simulations. Based on the complex active site model, the native active sites for ten reactions in the benchmark set have been recapitulated by the revised ProdaMatch, and the RMSDs of the matched TS from those in the crystal structures are less than 1.0 Å, which is much better than the results obtained by the minimal active site model. The minimal active site model is used by RosettaMatch [15], where the number of active site residues is ≤ 4 so as to avoid potential combinatorial explosion in matching. The number of active site residues in the complex active site model is much larger than that in the minimal active site model, but the combinatorial explosion problem was avoided by the revised version of ProdaMatch. To avoid combinatorial explosion in the revised version, catalytic geometric constraints between the catalytic residues and the TS and the side chain conformations of the catalytic residues are determined by the quasi-Newton, direction-based continuous optimization algorithm [34] and some effective pruning strategies for matching were developed herein. Furthermore, the side chain repacking calculation results for seven scaffolds whose TS positions were located by ProdaMatch were perfectly reproduced, with 91% of the hydrogen bonds in the active sites recovered and the calculated binding energies were comparable to those from the crystal structures.

Because more residues are included in the complex active site model for matching when compared with the minimal active site model, a better active site preorganization in the final design is achieved. Nonetheless, the complex model may be restricted in situations where only a few scaffolds can afford the complex active site model for a target reaction because the number of available structures in the PDB is limited. We have investigated this problem by using scaffold selection for hydrolytic reactions of PNPA and cephalexin based on the classic catalytic triad motif and one more flexible catalytic triad-based motif. The scaffold library constructed contained 1,491 proteins, which were selected from the intersection set of the PDB and the enzyme structure database CSA, and the structures in the scaffolds were diverse and the sequence homology between different structures was controlled. Richter *et al*. [20] constructed a minimal active site model for the hydrolytic reaction of an ester. The catalytic motif used in their model is composed of a cysteine-histidine dyad and a backbone amide group to stabilize the oxyanion of the TS. Although the primitive esterase was generated after multiple cycles of directed evolution, the activity of the designed esterase was found to be very low when compared with that of the native hydrolase. This was caused by the engineered Cys-His dyad always adopting different conformations from that in the computational design. In the native active sites of hydrolases, the classic catalytic triad was used as the nucleophile, and two backbone amide groups formed hydrogen bonds with the oxyanion of the TS and an additional backbone amide group formed a hydrogen bond with the catalytic triad Asp to facilitate its preorganization. By virtue of this classic complex active site model, scaffolds were selected by the revised ProdaMatch for the hydrolytic reaction of PNPA from the scaffold library constructed in this work. Eighty scaffolds were identified to afford the classic catalytic triad motif and four scaffolds were found to be native enzymes for the hydrolysis of PNPA. These results indicate that the revised ProdaMatch is able to anchor the complex active site model onto different scaffolds accurately.

The classic catalytic triad motif based active site is easy to pre-organize as three backbone hydrogen-bonding donors are used to stabilize the oxyanion of the TS and the catalytic triad aspartate. The low conformation flexibility of the backbone hydrogen bond donors greatly reduces the number of scaffolds for substrates with complex structures. This was confirmed by the scaffold selection results obtained by ProdaMatch for the hydrolysis of cephalexin based on the same catalytic motif as that for PNPA; note that cephalexin is a much more complex structure than PNPA. Only three scaffolds were identified for the hydrolysis of cephalexin when compared with the 80 scaffolds for PNPA. Native hydrolases use side chain hydrogen-bonding groups to stabilize the oxyanion and the catalytic triad Asp, and removal of the side chain oxyanion-stabilizing hydrogen bond was confirmed to dramatically reduce the catalytic efficacy of the native hydrolases [54]. Richter *et al*. [20] proposed in their esterase design to use one backbone NH group and one side chain group to stabilize the oxyanion. Rajagopalan *et al*. [42] used an additional arginine to stabilize the serine-containing catalytic triad aspartate for the hydrolytic reaction of the amide. Based on these facts, the complex active site model proposed for the hydrolysis of cephalexin is composed of more flexible residues and the complete catalytic motif is shown in Fig 5C. Compared with the classic catalytic triad motif, this catalytic motif has an increase in the number of conformational degrees of freedom. The scaffold selection results yielded 40 scaffolds that can afford the flexible active site model for the hydrolysis of cephalexin, and among them one scaffold (i.e., 1mpx) is the native α-amino acid ester hydrolase from *X*. *citri*, which catalyzes the hydrolysis and synthesis of cephalexin. Thus, the revised ProdaMach has reproduced the native active sites of 1mpx and ranked the native match high among all identified matches. Moreover, the flexible active site in 1mpx in the crystal structure is further stabilized by two additional residues, Tyr222 and Trp465, which form hydrogen bonds with Tyr82 and Glu309, respectively. These observations indicate that a preorganized active site is the origin of the catalytic power of enzyme catalysis, and active site preorganization modeling should aid *de novo* enzyme design significantly.

## Supporting Information

S1 Fig Overview of the matching process in ProdaMatch (Figure 2 in [26]). (TIF)

S2 Fig Complex active site model for 1dqx. The matching residues are classified into three groups: the primary catalytic residues are shown in blue, the residues interacting with catalytic residues are shown in pink, and the residues stabilizing the TS model are shown in light green. The number around the dashed line is the distance for hydrogen bond. (The same in S3–S9 Figs).
(TIF)

S3 Fig Complex active site model for 1h2j. (TIF)

S4 Fig Complex active site model for 1ney. (TIF)

S5 Fig Complex active site model for 1oex. (TIF)

S6 Fig Complex active site model for 1p6o. (TIF)

S7 Fig Complex active site model for 3vgc. (TIF)

S8 Fig Complex active site model for 4fua. (TIF)

S9 Fig Complex active site model for 6cpa. (TIF)

S10 Fig Superposition of native and predicted active sites for 1dqx. The transition states are shown in ball and stick model, and the active site residues in stick model. Atoms O, N, and C in crystal structures are colored in red, teal, and gray, respectively. The matched structures based on complex active site model are shown in pink. (The same in S11–S17 Figs).
(TIF)

S11 Fig Superposition of native and predicted active sites for 1h2j. (TIF)

S12 Fig Superposition of native and predicted active sites for 1ney. (TIF)

S13 Fig Superposition of native and predicted active sites for 1oex. (TIF)

S14 Fig Superposition of native and predicted active sites for 1p6o. (TIF)

S15 Fig Superposition of native and predicted active sites for 3vgc. (TIF)

S16 Fig Superposition of native and predicted active sites for 4fua. (TIF)

S17 Fig Superposition of native and predicted active sites for 6cpa. (TIF)

S1 Table List of PDB entries used as scaffolds in scaffold library. (DOC)

S2 Table Matching parameters for 1c2t based on complex active site model. (DOC)

S3 Table Matching parameters for 1c2t based on minimal active site model. (DOC)

S4 Table Matching parameters for 1dqx based on complex active site model. (DOC)

S5 Table Matching parameters for 1dqx based on minimal active site model. (DOC)

S6 Table Matching parameters for 1h2j based on complex active site model. (DOC)

S7 Table Matching parameters for 1h2j based on minimal active site model. (DOC)

S8 Table Matching parameters for 1jcl based on complex active site model. (DOC)

S9 Table Matching parameters for 1jcl based on minimal active site model. (DOC)

S10 Table Matching parameters for 1ney based on complex active site model. (DOC)

S11 Table Matching parameters for 1ney based on minimal active site model. (DOC)

S12 Table Matching parameters for 1oex based on complex active site model. (DOC)

S13 Table Matching parameters for 1oex based on minimal active site model. (DOC)

S14 Table Matching parameters for 1p6o based on complex active site model. (DOC)

S15 Table Matching parameters for 1p6o based on minimal active site model. (DOC)

S16 Table Matching parameters for 3vgc based on complex active site model. (DOC)

S17 Table Matching parameters for 3vgc based on minimal active site model. (DOC)

S18 Table Matching parameters for 4fua based on complex active site model. (DOC)

S19 Table Matching parameters for 4fua based on minimal active site model. (DOC)

S20 Table Matching parameters for 6cpa based on complex active site model. (DOC)

S21 Table Matching parameters for 6cpa based on minimal active site model. (DOC)

S22 Table RMS deviations of residues in native matches and CPU running time of ProdaMatch for ten scaffolds. (DOC)

S23 Table Hydrogen bonding recapitulation results for seven scaffolds based on different active site models. (DOC)

S24 Table Matching parameters for PNPA based on classic catalytic triad motif. (DOC)

S25 Table Matching parameters for cephalexin based on classic catalytic triad motif. (DOC)

S26 Table Matching parameters for cephalexin based on flexible catalytic triad motif. (DOC)
