# Supplementary figures and images for: Use of an Improved Matching Algorithm to Select Scaffolds for Enzyme Design Based on a Complex Active Site Model
Source: PLoS One. 2016 May 31;11(5):e0156559. doi: 10.1371/journal.pone.0156559 (PMC4887040; doi:10.1371/journal.pone.0156559)

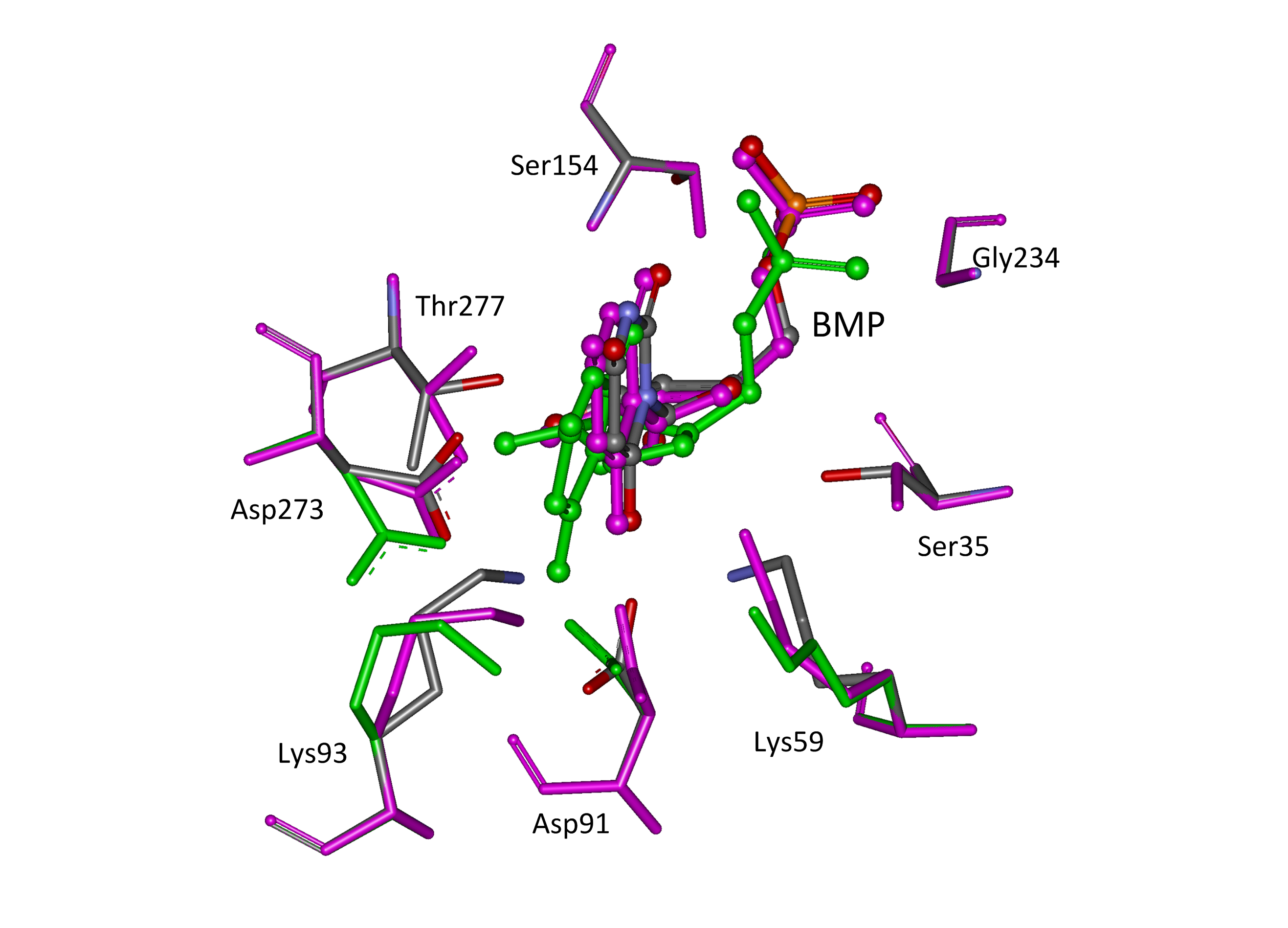

Supplement: S10 Fig — The transition states are shown in ball and stick model, and the active site residues in stick model. Atoms O, N, and C in crystal structures are colored in red, teal, and gray, respectively. The matched structures based on complex active site model are shown in pink. (The same in S11–S17 Figs). (TIF) [file pone.0156559.s010.tif]

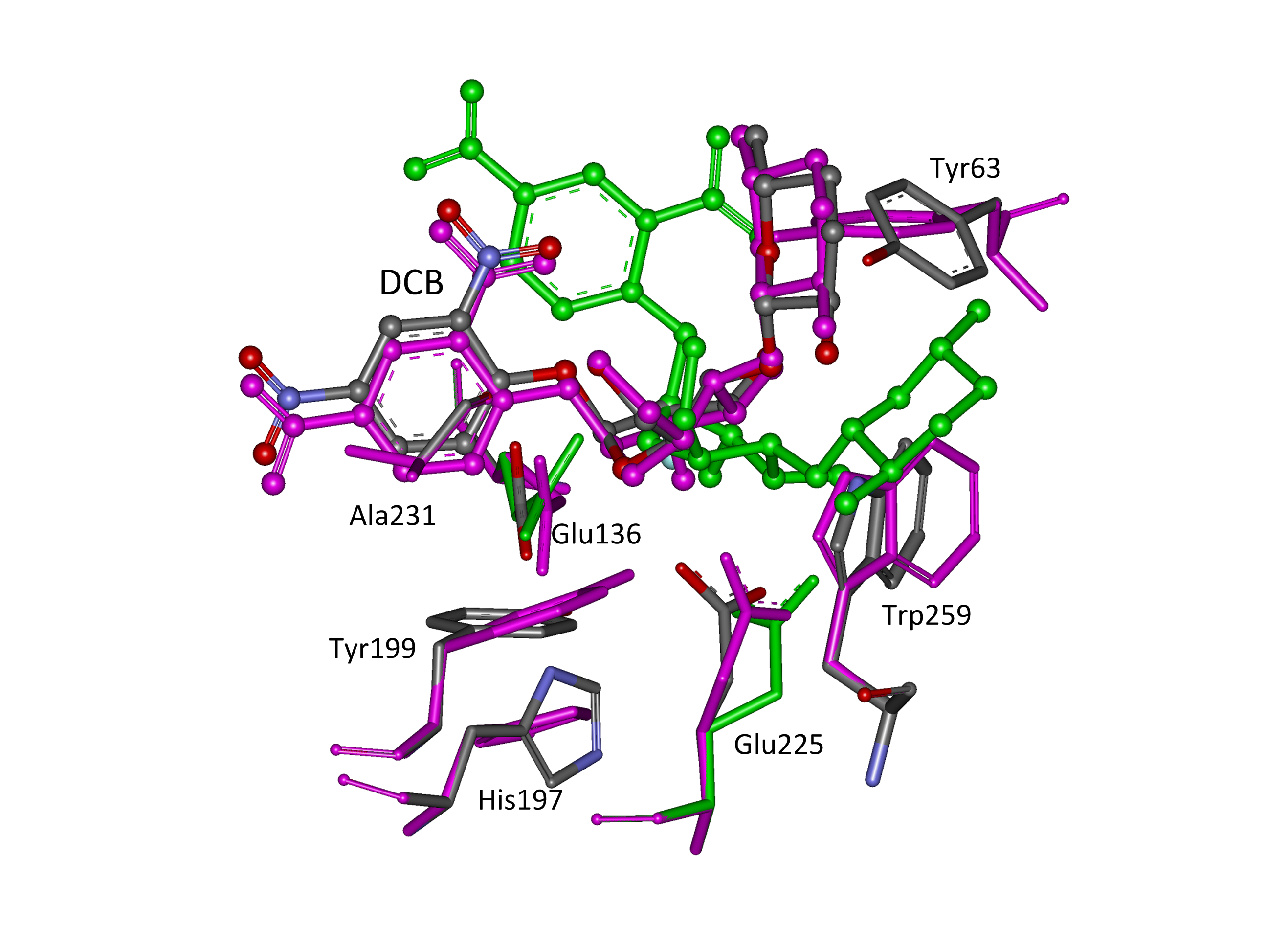

Supplement: S11 Fig — (TIF) [file pone.0156559.s011.tif]

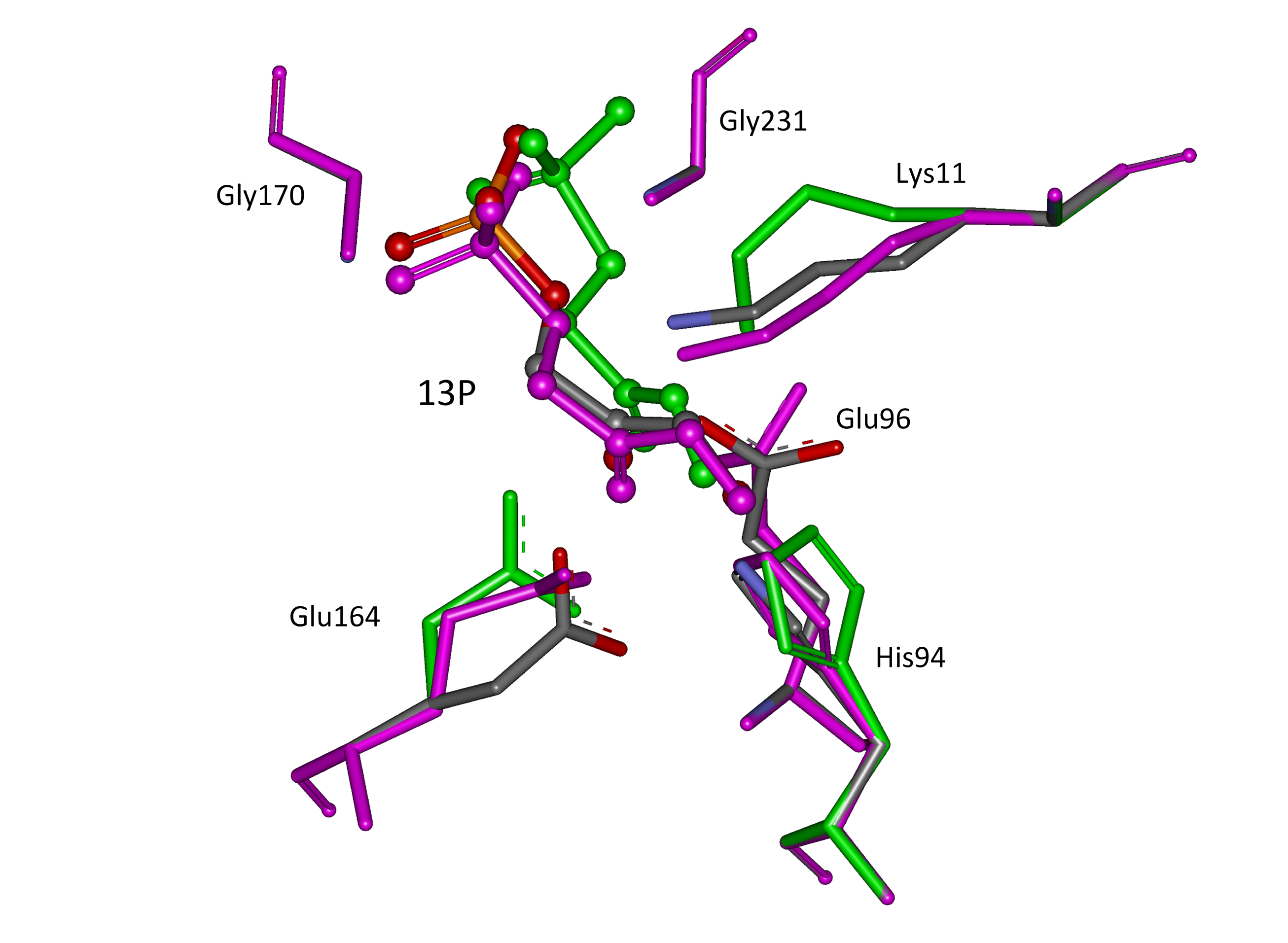

Supplement: S12 Fig — (TIF) [file pone.0156559.s012.tif]

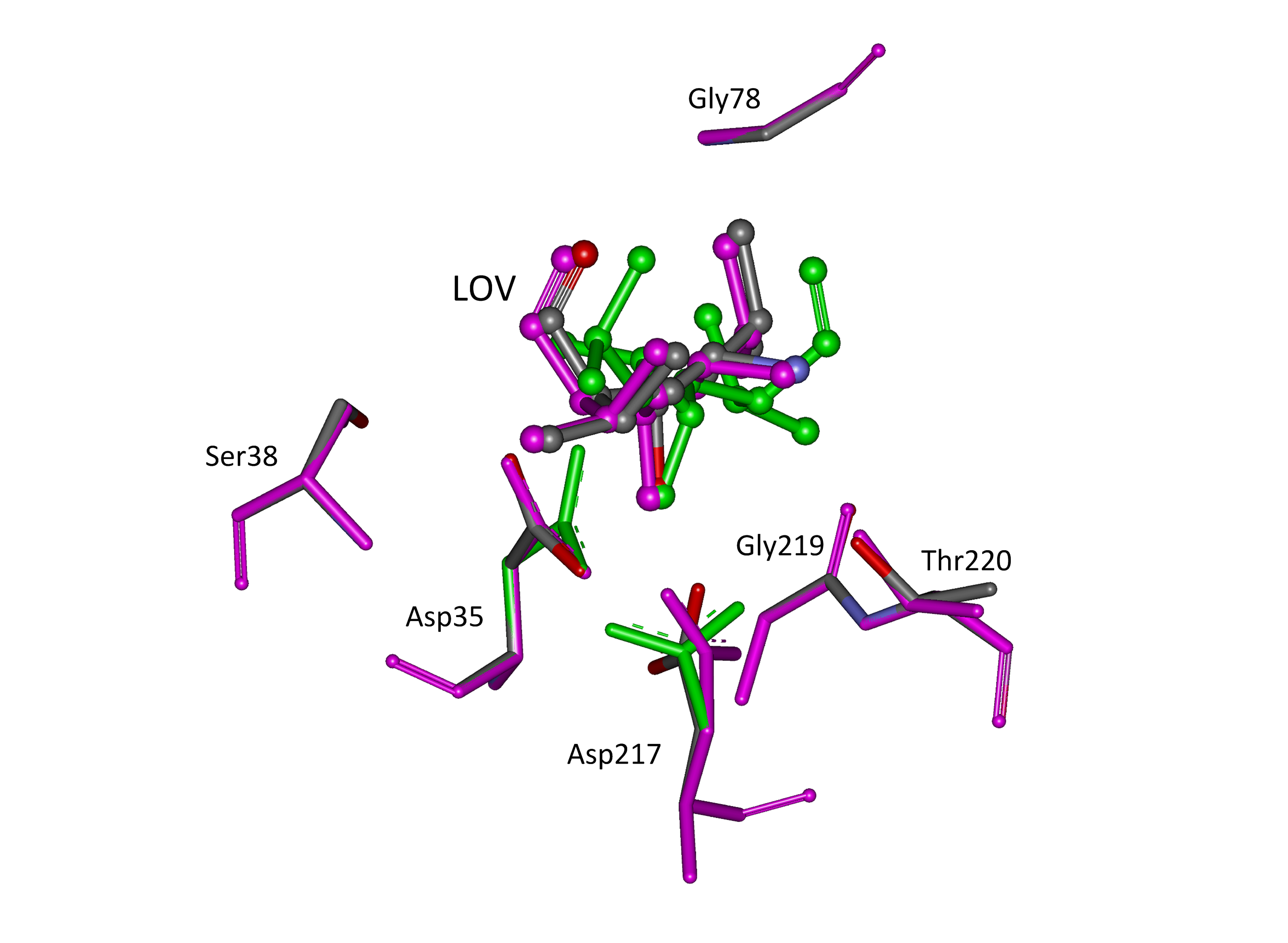

Supplement: S13 Fig — (TIF) [file pone.0156559.s013.tif]

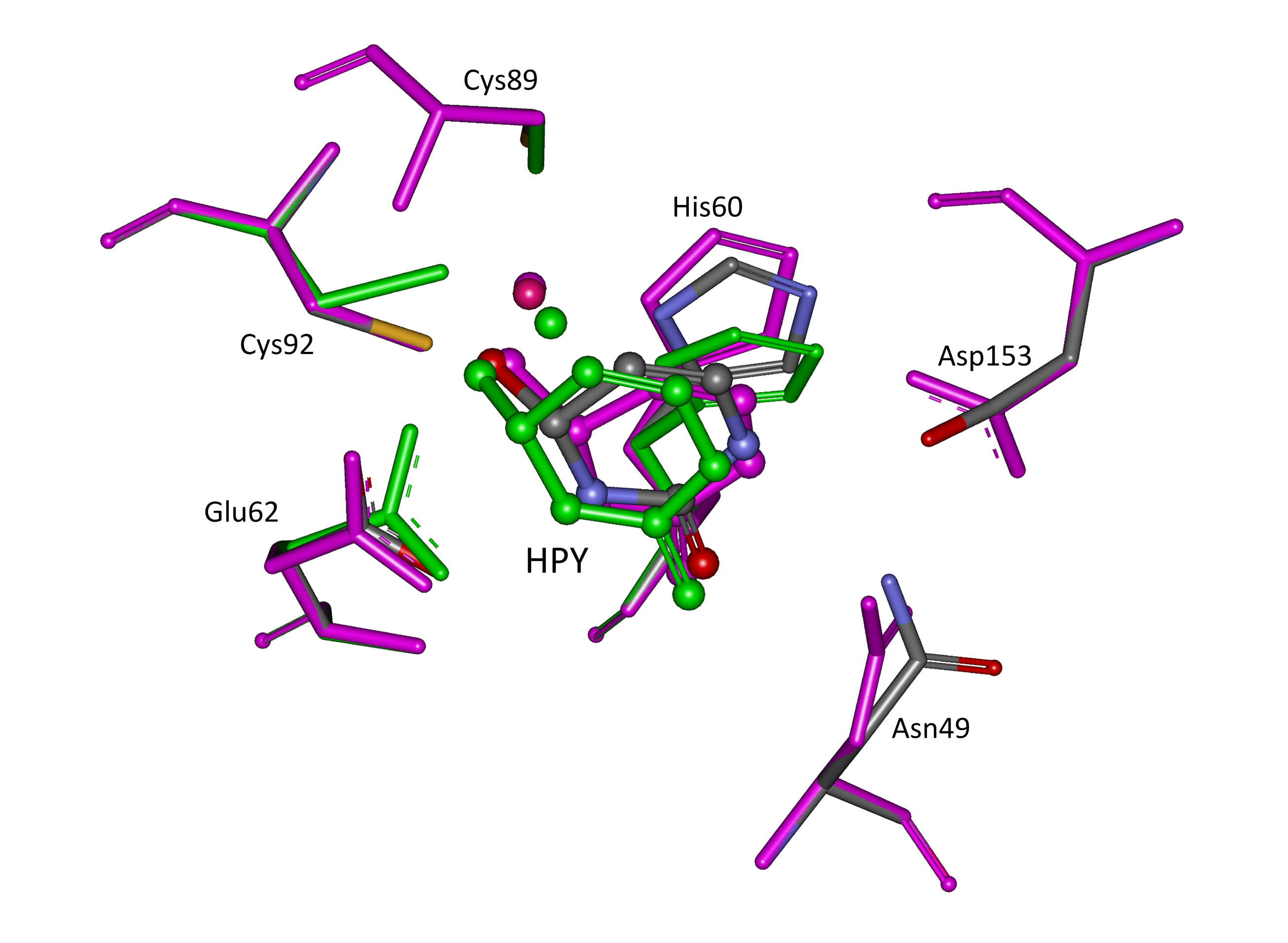

Supplement: S14 Fig — (TIF) [file pone.0156559.s014.tif]

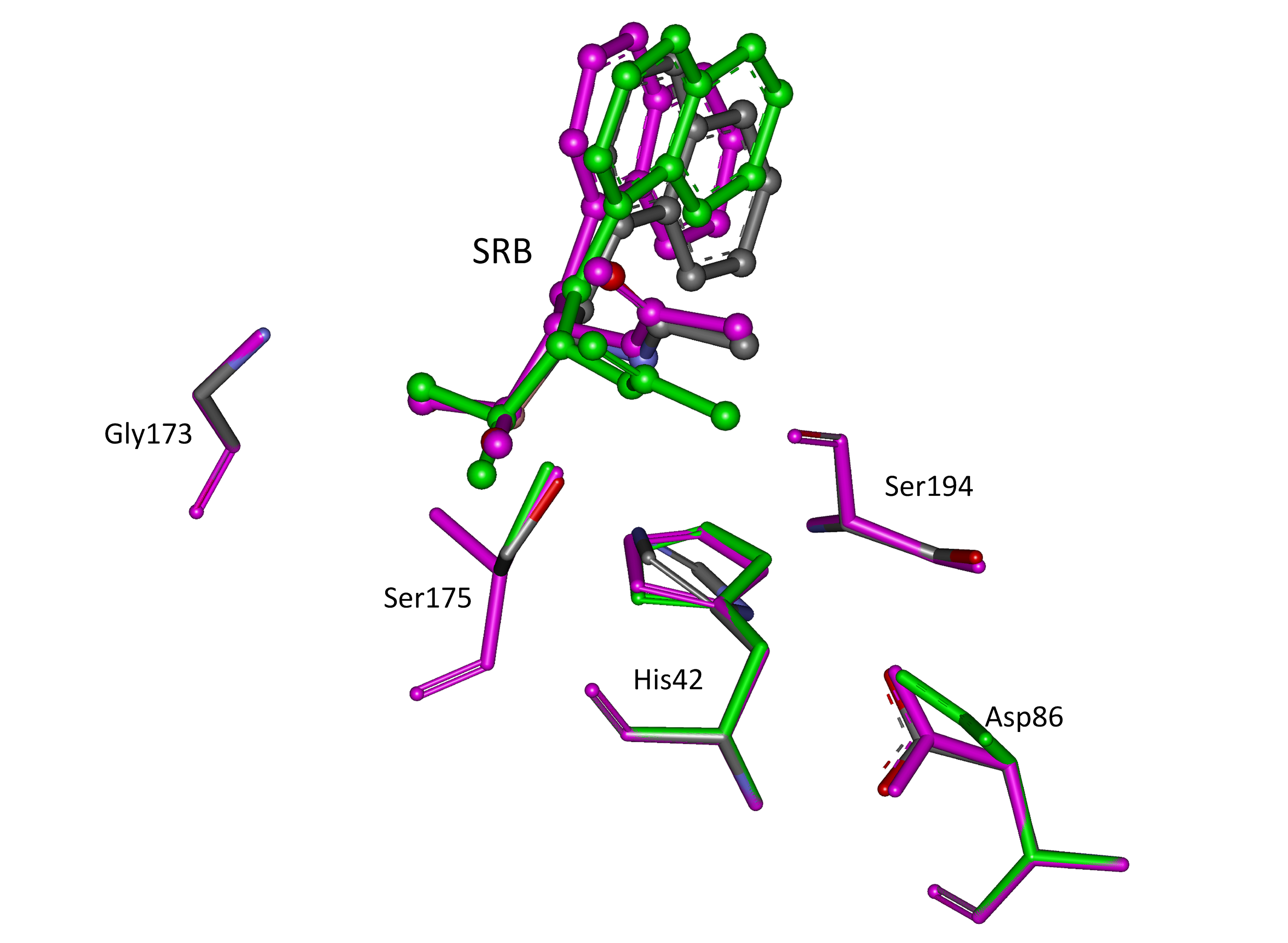

Supplement: S15 Fig — (TIF) [file pone.0156559.s015.tif]

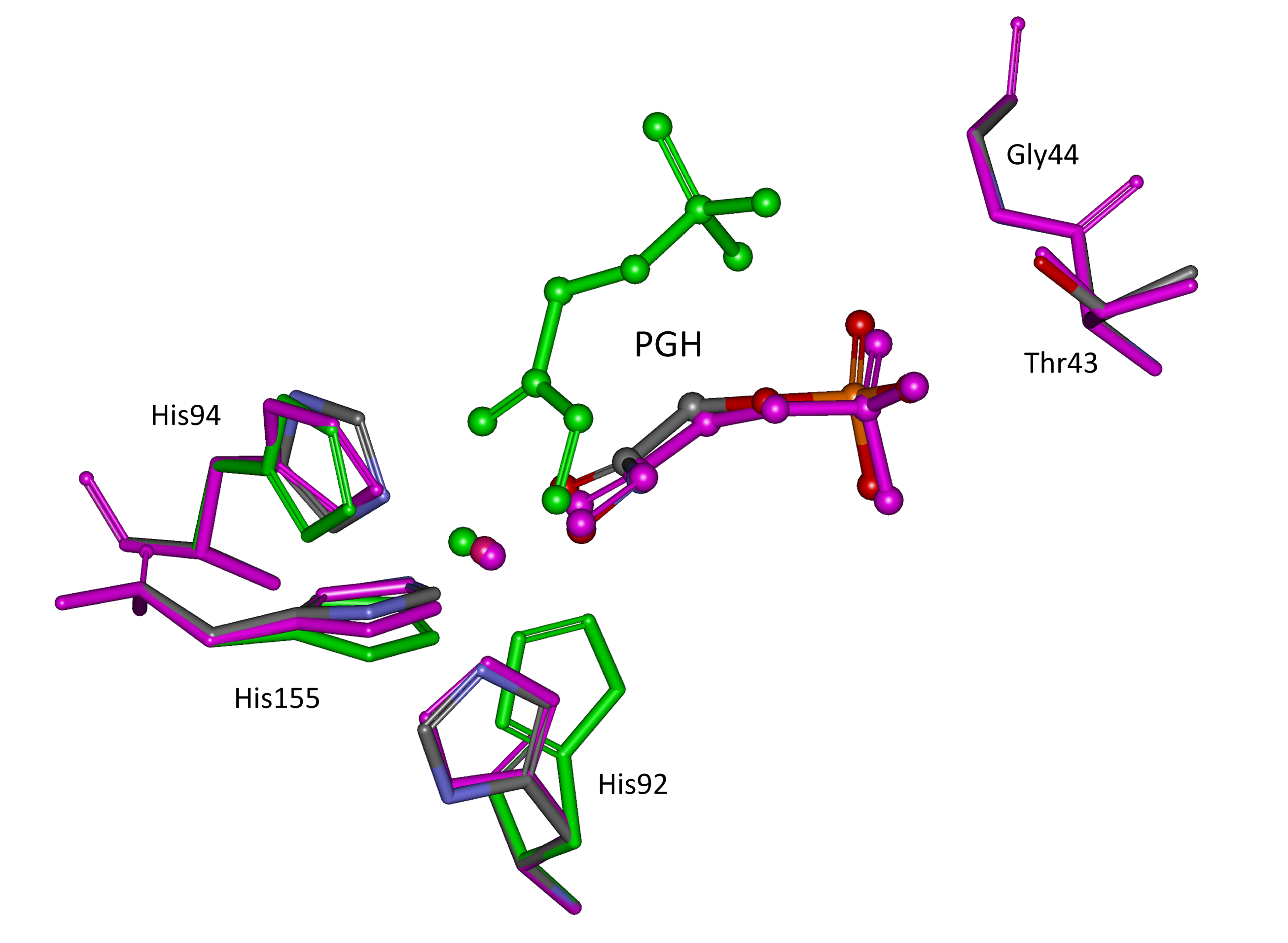

Supplement: S16 Fig — (TIF) [file pone.0156559.s016.tif]

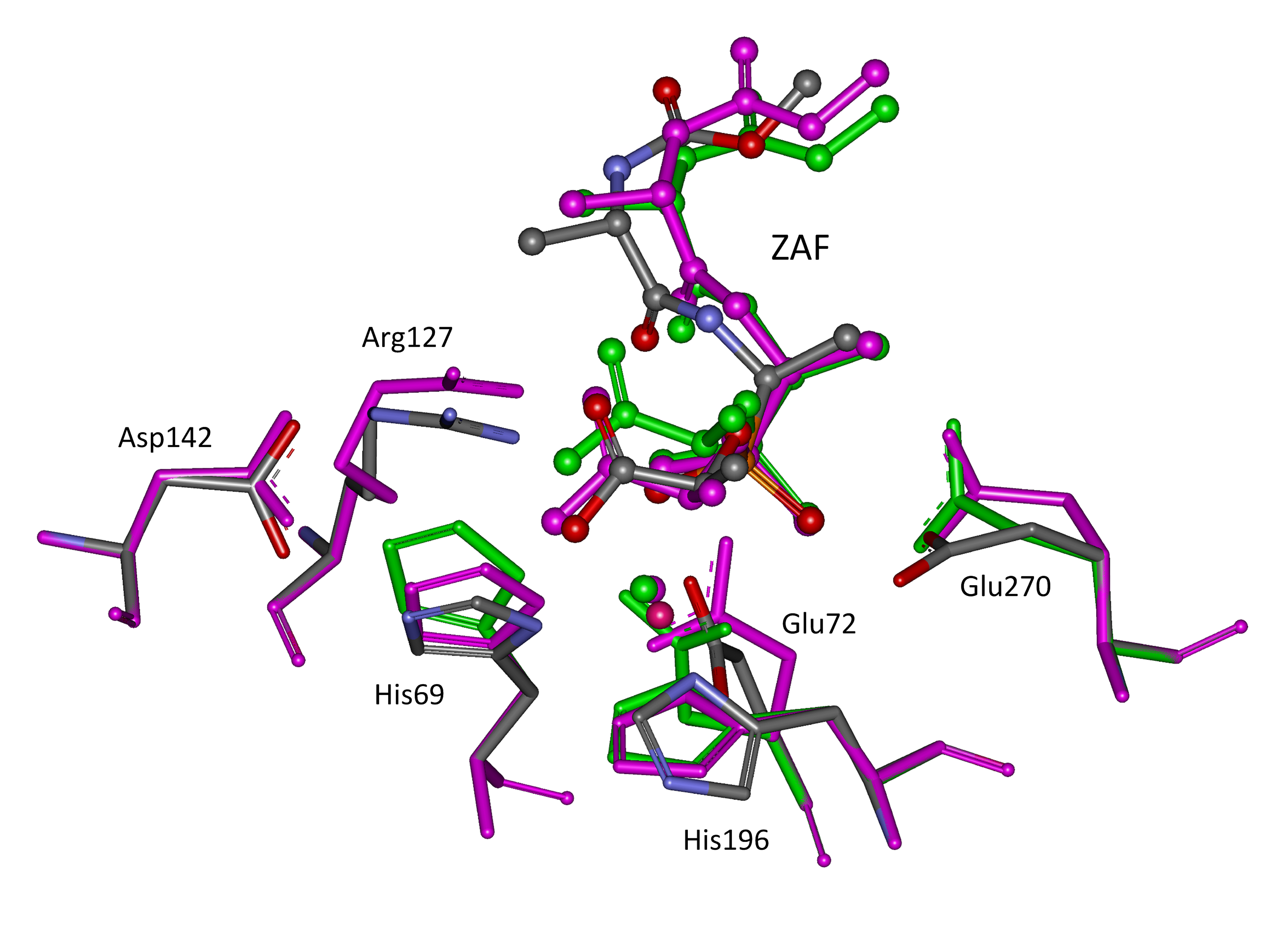

Supplement: S17 Fig — (TIF) [file pone.0156559.s017.tif]
