# Supplementary material for: Use of an Improved Matching Algorithm to Select Scaffolds for Enzyme Design Based on a Complex Active Site Model
Source: PLoS One. 2016 May 31;11(5):e0156559. doi: 10.1371/journal.pone.0156559 (PMC4887040; doi:10.1371/journal.pone.0156559)
Supplement: S1 Table — (DOC) [file pone.0156559.s018.doc]

**S1 Table. List of PDB entries used as scaffolds in scaffold library.**

| 12as | 1a05 | 1a0c | 1a0e | 1a0i | 1a2o | 1a47 | 1a4i | 1a59 | 1a5z |
| --- | --- | --- | --- | --- | --- | --- | --- | --- | --- |
| 1aa6 | 1ad3 | 1aj8 | 1al8 | 1amu | 1ao0 | 1aqj | 1aqm | 1aui | 1ax4 |
| 1axk | 1ayx | 1azw | 1b1y | 1b5p | 1b65 | 1b6c | 1b6g | 1b7g | 1b80 |
| 1b8a | 1b9h | 1bbu | 1bdg | 1bg6 | 1bi9 | 1bif | 1bou | 1bqg | 1bs0 |
| 1bs2 | 1bsl | 1bx1 | 1bx4 | 1bxb | 1bxc | 1bxk | 1by8 | 1bzl | 1c3c |
| 1c4k | 1c4z | 1c7g | 1c7q | 1c9w | 1ccw | 1cf2 | 1cg2 | 1ci9 | 1cib |
| 1cki | 1cl0 | 1cm8 | 1cnz | 1cru | 1ct9 | 1czi | 1d1t | 1d6f | 1d6m |
| 1d6s | 1d8w | 1d9q | 1db3 | 1ddg | 1dfa | 1di1 | 1dj2 | 1dj3 | 1dki |
| 1dl5 | 1dlj | 1dmh | 1dos | 1dpg | 1dpj | 1dqs | 1duv | 1dxy | 1e0t |
| 1e1m | 1e1o | 1e2k | 1e3i | 1e4e | 1e4i | 1e5m | 1e5q | 1e6u | 1e8g |
| 1e93 | 1e94 | 1e9i | 1ebd | 1ebf | 1ec7 | 1edz | 1ei5 | 1ejd | 1ems |
| 1eno | 1eov | 1ep3 | 1eq2 | 1eqr | 1esw | 1eud | 1euh | 1euz | 1evy |
| 1ez0 | 1ez4 | 1ezr | 1f05 | 1f0y | 1f1j | 1f20 | 1f2d | 1f2j | 1f34 |
| 1f3l | 1f52 | 1f5a | 1f60 | 1f6d | 1f6w | 1f76 | 1f82 | 1f8m | 1f8w |
| 1f9v | 1fec | 1fg5 | 1fl2 | 1fmv | 1fp1 | 1fp2 | 1frb | 1fur | 1fwy |
| 1g0d | 1g55 | 1g5a | 1g6q | 1g6s | 1g8m | 1g8p | 1ga6 | 1gad | 1gcy |
| 1gk9 | 1gkm | 1goj | 1got | 1gp6 | 1gpm | 1gq2 | 1gsa | 1gtk | 1gv0 |
| 1gwu | 1gyt | 1gz6 | 1h3f | 1h6d | 1hd7 | 1hdh | 1hk8 | 1hm9 | 1hp1 |
| 1hqs | 1hsk | 1hso | 1hw4 | 1hxp | 1hye | 1hyu | 1i0d | 1i24 | 1i32 |
| 1i7q | 1i8t | 1iat | 1igw | 1ihu | 1ii2 | 1iic | 1iip | 1io7 | 1itx |
| 1itz | 1iu4 | 1iuq | 1ivh | 1iy7 | 1iye | 1iyk | 1j0b | 1j0h | 1j1b |
| 1j20 | 1j33 | 1j3b | 1j4a | 1j6u | 1j70 | 1j8u | 1j93 | 1ja1 | 1jbq |
| 1jdc | 1jdw | 1jfb | 1jhd | 1ji1 | 1jil | 1jkm | 1jl0 | 1jof | 1jqi |
| 1js1 | 1jub | 1jvb | 1k28 | 1k75 | 1k89 | 1k92 | 1k9o | 1kbi | 1kbj |
| 1kbv | 1kcb | 1kcz | 1kf6 | 1kfg | 1kfi | 1khb | 1kjq | 1kk0 | 1kl7 |
| 1kmj | 1kol | 1kp8 | 1krh | 1kwp | 1kxq | 1kyz | 1l1f | 1l1l | 1l5w |
| 1l6j | 1l6s | 1l7d | 1l8k | 1l9x | 1lbq | 1lci | 1lf2 | 1lfk | 1li4 |
| 1lii | 1ll7 | 1lqa | 1lqt | 1lth | 1ltq | 1lvo | 1lwj | 1m15 | 1m7s |
| 1mas | 1mdb | 1mdo | 1mj5 | 1ml4 | 1mla | 1mlw | 1moq | 1mpx | 1muw |
| 1mw9 | 1mx3 | 1mxr | 1mzy | 1n1b | 1n2t | 1n40 | 1n7o | 1nj1 | 1nm8 |
| 1np7 | 1nr6 | 1nu5 | 1nuy | 1nvm | 1nw6 | 1nxu | 1nyl | 1o4s | 1o6i |
| 1o7x | 1o88 | 1o94 | 1o98 | 1o9j | 1oc4 | 1odm | 1odt | 1ogp | 1ohl |
| 1okg | 1omo | 1on3 | 1onf | 1onw | 1oq9 | 1or0 | 1ovm | 1owl | 1ox6 |
| 1p3d | 1p4c | 1p5g | 1p6x | 1p7t | 1pa2 | 1pam | 1pb1 | 1pbg | 1pe9 |
| 1peg | 1pfz | 1pix | 1pj3 | 1pj9 | 1pjr | 1pjs | 1pkx | 1po5 | 1ps1 |
| 1ps9 | 1pv8 | 1pwh | 1pyf | 1pz3 | 1q1q | 1q5d | 1q5m | 1q5n | 1q6z |
| 1q7e | 1q8y | 1qak | 1qd1 | 1qdl | 1qe3 | 1qfz | 1qgj | 1qh4 | 1qj5 |
| 1qk1 | 1qmg | 1qo5 | 1qqj | 1qwl | 1r0v | 1r3s | 1r5b | 1r6w | 1r76 |
| 1r9o | 1ra0 | 1req | 1rf6 | 1rg9 | 1rgy | 1rgz | 1rjw | 1rkd | 1rqb |
| 1rqq | 1ru4 | 1rxt | 1ry2 | 1ryd | 1s1p | 1s70 | 1sc6 | 1sgv | 1shz |
| 1smk | 1snr | 1snz | 1sov | 1svu | 1syy | 1sz2 | 1t10 | 1t1u | 1t2a |
| 1t2f | 1t3i | 1t4b | 1t5c | 1t7q | 1t90 | 1tb4 | 1tel | 1tjv | 1txg |
| 1tzj | 1tzs | 1u2k | 1u3d | 1u3u | 1u3w | 1u6r | 1u8f | 1uas | 1ubv |
| 1ued | 1uf5 | 1ujn | 1ujq | 1umd | 1uok | 1uou | 1uqt | 1us0 | 1uuf |
| 1uxj | 1uyp | 1v25 | 1v8b | 1v8k | 1v9p | 1vb3 | 1vdc | 1vem | 1vi2 |
| 1vl2 | 1vlc | 1vlo | 1vlv | 1vpe | 1vr6 | 1vrp | 1w0c | 1w0d | 1w23 |
| 1w27 | 1w32 | 1w5q | 1w6t | 1w6u | 1w85 | 1w8o | 1w9y | 1wch | 1wdk |
| 1wdp | 1wl4 | 1wle | 1wm1 | 1wos | 1wpw | 1wsr | 1wvf | 1wvg | 1wy2 |
| 1wyd | 1wzl | 1x0l | 1x0v | 1x13 | 1x1i | 1x54 | 1x7d | 1x9i | 1xah |
| 1xel | 1xfb | 1xk7 | 1xlm | 1xmc | 1xp3 | 1xql | 1xrs | 1xsm | 1xyz |
| 1y2m | 1y42 | 1y6b | 1y79 | 1y9a | 1ych | 1yfm | 1yis | 1ykf | 1ylh |
| 1yoe | 1ytm | 1yvg | 1yw9 | 1z41 | 1z5h | 1z8o | 1zai | 1zbu | 1zc0 |
| 1zcj | 1zcz | 1zlp | 1zmd | 1zmr | 1zod | 1zq1 | 1zxx | 1zy4 | 2a2a |
| 2a9d | 2aeb | 2akj | 2akz | 2apo | 2ash | 2aus | 2axq | 2ay1 | 2ayq |
| 2b3o | 2b4g | 2b9h | 2be9 | 2bfd | 2bgs | 2bh9 | 2bht | 2bi7 | 2bjf |
| 2bju | 2bmb | 2bmw | 2bo9 | 2bp7 | 2bpo | 2buf | 2buj | 2bwp | 2bzl |
| 2bzr | 2c0r | 2c1h | 2c2n | 2c30 | 2c31 | 2c47 | 2c4e | 2c5a | 2c61 |
| 2c7s | 2c81 | 2c8j | 2c8n | 2cb5 | 2cca | 2cfo | 2cfv | 2cjz | 2cm2 |
| 2cmw | 2coi | 2cun | 2cxe | 2cxn | 2czc | 2d1f | 2d1s | 2d4a | 2dbq |
| 2dfd | 2dfi | 2dfv | 2dkv | 2dpl | 2dq0 | 2dq3 | 2dq4 | 2dw4 | 2dwc |
| 2dyu | 2e01 | 2e1z | 2e28 | 2e5v | 2e8y | 2e9l | 2eer | 2eja | 2ep7 |
| 2es4 | 2et6 | 2eu9 | 2ey4 | 2ez1 | 2f00 | 2fdv | 2fh7 | 2fok | 2fp3 |
| 2fpq | 2fst | 2fyf | 2fze | 2g24 | 2g36 | 2g76 | 2g8s | 2gcg | 2gdq |
| 2gf3 | 2gi3 | 2gk9 | 2gn0 | 2go1 | 2gp6 | 2gq1 | 2gru | 2gry | 2h0v |
| 2h12 | 2h4v | 2h58 | 2hak | 2hc9 | 2heh | 2hej | 2hel | 2hg2 | 2hgs |
| 2hhp | 2hjh | 2hro | 2hsa | 2hw6 | 2hwg | 2hxt | 2i1y | 2i4g | 2i4l |
| 2i5p | 2i6l | 2i6u | 2i87 | 2iag | 2ib8 | 2ifc | 2ify | 2inf | 2inr |
| 2isq | 2iu8 | 2iv0 | 2iw2 | 2iwz | 2ix4 | 2ix5 | 2iz1 | 2izr | 2j07 |
| 2j0i | 2j1q | 2j4d | 2j66 | 2j6i | 2j6l | 2j78 | 2j7t | 2j8g | 2j90 |
| 2j91 | 2ja2 | 2-Jan | 2jbv | 2jc3 | 2jc6 | 2jif | 2jjk | 2jjn | 2jkb |
| 2nlk | 2ntp | 2nu8 | 2nxw | 2nya | 2o0b | 2o2c | 2o2e | 2o2i | 2o36 |
| 2o3e | 2o3j | 2o4c | 2o5r | 2o7r | 2o7s | 2o9p | 2oat | 2obv | 2oc3 |
| 2od2 | 2ode | 2oem | 2ofp | 2oh4 | 2ohh | 2olq | 2oo0 | 2oo8 | 2ort |
| 2otn | 2p0c | 2p0r | 2p3e | 2p4q | 2p6x | 2p9t | 2pa6 | 2pan | 2pid |
| 2poc | 2psd | 2psn | 2psq | 2ptr | 2q28 | 2q3m | 2q3o | 2q3r | 2q3z |
| 2q4e | 2q4h | 2q4w | 2q7w | 2q8n | 2qae | 2qcv | 2qep | 2qgh | 2qj3 |
| 2qjf | 2qkx | 2qn0 | 2qr7 | 2quy | 2r0i | 2r11 | 2r2j | 2r3a | 2r4f |
| 2r7b | 2r8o | 2r9f | 2rcc | 2rdu | 2reo | 2rhs | 2rkb | 2shp | 2tod |
| 2toh | 2uuq | 2uwf | 2uxw | 2v09 | 2v1p | 2v3a | 2v3z | 2v40 | 2v5h |
| 2v5q | 2v65 | 2v6a | 2v6b | 2v6c | 2v7o | 2v7q | 2v7y | 2v8q | 2vag |
| 2vba | 2vcy | 2vd4 | 2vd5 | 2ve3 | 2vef | 2vhd | 2vig | 2vjq | 2vk8 |
| 2vp8 | 2vu1 | 2vvm | 2vwi | 2vx3 | 2vxo | 2vz6 | 2w0b | 2w20 | 2w2d |
| 2w2n | 2w37 | 2w4o | 2w5f | 2w8n | 2wbi | 2wdq | 2we5 | 2wel | 2wfp |
| 2wgh | 2whz | 2wlr | 2wlt | 2wm5 | 2woj | 2wq8 | 2wqd | 2wqm | 2wsk |
| 2wtb | 2wu8 | 2wu9 | 2wvg | 2wxu | 2wy8 | 2wzb | 2x06 | 2x5o | 2x6t |
| 2x75 | 2x7j | 2xap | 2xb6 | 2xdw | 2xfg | 2xgz | 2xhl | 2xij | 2xkr |
| 2xq0 | 2xqr | 2xrw | 2xsn | 2xsx | 2xtz | 2y3z | 2y65 | 2y7j | 2yab |
| 2ybx | 2ycf | 2yfh | 2yfi | 2yfq | 2ywb | 2ywg | 2yxn | 2yxx | 2yyy |
| 2z04 | 2z26 | 2z3z | 2z61 | 2zad | 2zbw | 2zbx | 2zdh | 2zfi | 2zj3 |
| 2zkj | 2zmd | 2zpu | 2zsi | 2zu2 | 2zvi | 2zwu | 3a04 | 3a2q | 3a32 |
| 3a51 | 3a74 | 3a99 | 3aal | 3ab1 | 3ado | 3afh | 3afi | 3aii | 3aj7 |
| 3alo | 3aqi | 3asa | 3atv | 3b12 | 3b1u | 3b2t | 3b3d | 3b4x | 3b6r |
| 3b6u | 3b6v | 3b7o | 3ba1 | 3bfn | 3bg9 | 3bh7 | 3bhg | 3bkb | 3bmx |
| 3bon | 3bow | 3box | 3bpt | 3bul | 3buv | 3c17 | 3c7a | 3ce6 | 3cg7 |
| 3clh | 3cmc | 3cog | 3cov | 3cq0 | 3csu | 3cui | 3cx5 | 3cx8 | 3cyv |
| 3czh | 3d2f | 3d3l | 3d4p | 3d4u | 3d5t | 3d9d | 3dbg | 3dc4 | 3ddn |
| 3dg8 | 3dgh | 3dgz | 3djl | 3dk9 | 3dko | 3dva | 3dwb | 3dwg | 3dyd |
| 3e04 | 3e0m | 3e2t | 3e4c | 3e77 | 3e7g | 3e7o | 3e7w | 3e9k | 3ea4 |
| 3ecr | 3eg4 | 3egg | 3ego | 3ehb | 3ejx | 3elf | 3emc | 3err | 3etj |
| 3eua | 3ewm | 3exe | 3eya | 3f3s | 3f9m | 3fce | 3fe1 | 3fe3 | 3ff1 |
| 3fg1 | 3fgc | 3fhr | 3fie | 3fjo | 3fju | 3fk4 | 3flk | 3fmu | 3fpc |
| 3fpl | 3fq8 | 3fr7 | 3fsl | 3fst | 3fy4 | 3g4d | 3gb9 | 3gbj | 3gc2 |
| 3gd5 | 3gdo | 3gdq | 3ge3 | 3gfb | 3ggf | 3gh0 | 3gl1 | 3glq | 3goa |
| 3goq | 3gp0 | 3gqb | 3gr4 | 3gr7 | 3gtd | 3gvi | 3gvp | 3gzd | 3gzy |
| 3h0l | 3h0p | 3h1d | 3h42 | 3h49 | 3h4j | 3h4s | 3h5q | 3h7r | 3h7u |
| 3h8g | 3h9c | 3h9e | 3hbg | 3hf1 | 3hgr | 3hhp | 3hid | 3hja | 3hjb |
| 3hm8 | 3hmp | 3hnc | 3hng | 3ho9 | 3hqn | 3ht5 | 3hwc | 3hzu | 3i28 |
| 3i2k | 3i33 | 3i3t | 3i6u | 3i99 | 3iar | 3iau | 3ibd | 3icf | 3ids |
| 3ifs | 3ihj | 3ii0 | 3ik0 | 3im9 | 3iml | 3inn | 3io3 | 3ip4 | 3ipl |
| 3iqi | 3irm | 3ish | 3itj | 3iu0 | 3iu1 | 3ivy | 3ix6 | 3jru | 3jsl |
| 3jtm | 3ju4 | 3ju5 | 3ju8 | 3jz6 | 3jze | 3k35 | 3k3p | 3k65 | 3k8z |
| 3k92 | 3k96 | 3k9v | 3ka0 | 3kb9 | 3kcg | 3kd9 | 3kdn | 3kki | 3kld |
| 3kn6 | 3kp1 | 3kr6 | 3ksk | 3kul | 3kvw | 3kyh | 3kzn | 3l24 | 3l6b |
| 3l6c | 3l8c | 3l9w | 3lb8 | 3ld6 | 3ldo | 3lfu | 3ljk | 3lk7 | 3lm5 |
| 3lms | 3ln3 | 3lpf | 3lq1 | 3lre | 3lvm | 3lwb | 3lxm | 3lzw | 3m00 |
| 3m4y | 3m5u | 3m6i | 3m83 | 3mbd | 3mca | 3mdm | 3mdy | 3mfr | 3mhs |
| 3mog | 3mpi | 3mtg | 3mtl | 3mvi | 3mwd | 3mz0 | 3n0g | 3n2b | 3n37 |
| 3n3r | 3n58 | 3n75 | 3n80 | 3nc3 | 3ng0 | 3nr9 | 3nvs | 3nyd | 3nyt |
| 3o23 | 3o47 | 3o4p | 3o8j | 3oet | 3ofm | 3ohs | 3okf | 3olh | 3olm |
| 3oml | 3ond | 3oz7 | 3ozu | 3p14 | 3p1a | 3p26 | 3p5p | 3p86 | 3p96 |
| 3pao | 3pbh | 3pd6 | 3pdk | 3pdx | 3pgj | 3piu | 3pki | 3pm0 | 3pm6 |
| 3pmo | 3pp8 | 3ppm | 3pqa | 3pqe | 3prh | 3pyf | 3pzr | 3q1k | 3q3v |
| 3q52 | 3q7e | 3q98 | 3q9o | 3qah | 3qan | 3qbe | 3qd2 | 3qe2 | 3qe3 |
| 3qfa | 3qft | 3qfu | 3qkz | 3qm2 | 3qm3 | 3qml | 3qn3 | 3qne | 3qrv |
| 3qtg | 3qtp | 3qz1 | 3r0q | 3r31 | 3r38 | 3r44 | 3r5x | 3r7f | 3r7t |
| 3r8w | 3r9p | 3rde | 3rhj | 3rim | 3rjl | 3rlg | 3rlh | 3rm5 | 3rmj |
| 3rmt | 3ro8 | 3rr1 | 3rtk | 3ru6 | 3rv2 | 3s3e | 3s46 | 3s6d | 3s82 |
| 3s9v | 3s9z | 3sds | 3sg1 | 3sgz | 3sk0 | 3sk3 | 3slh | 3sls | 3smq |
| 3smt | 3sr7 | 3swe | 3swg | 3sxx | 3sz3 | 3sza | 3t4e | 3t57 | 3t5a |
| 3t5c | 3t7v | 3t8b | 3tac | 3tbf | 3tcm | 3tde | 3teg | 3tfj | 3tg0 |
| 3thi | 3tjz | 3tl2 | 3tlo | 3tnl | 3tpf | 3tqi | 3tqp | 3tqt | 3tsr |
| 3ttv | 3tug | 3tut | 3twl | 3tx1 | 3tx8 | 3ty4 | 3ty7 | 3tz6 | 3tzl |
| 3u06 | 3u28 | 3u3o | 3u9w | 3ubd | 3uc3 | 3uc4 | 3udb | 3udu | 3ue9 |
| 3ufx | 3ug7 | 3uh0 | 3uim | 3uiu | 3uko | 3ulk | 3umo | 3uq8 | 3utn |
| 3uto | 3uwd | 3uwl | 3uxo | 3v0s | 3v1y | 3v4z | 3v8d | 3v98 | 3vbb |
| 3vcy | 3vku | 3vmf | 3vmj | 3vn9 | 3voc | 3vpg | 3vsa | 3vv2 | 3zcw |
| 3zfd | 3zia | 3zq6 | 3ztv | 3zwc | 3zxw | 3zzh | 3zzm | 4a0m | 4a14 |
| 4a1n | 4a3q | 4a3r | 4a3s | 4a8t | 4acf | 4adm | 4aec | 4agu | 4aj9 |
| 4ajj | 4amu | 4amv | 4asi | 4aw2 | 4b9d | 4bbn | 4d9b | 4dd5 | 4dg5 |
| 4dng | 4dnx | 4dq1 | 4dq8 | 4dql | 4dr0 | 4dv8 | 4e01 | 4e0b | 4e37 |
| 4e3x | 4e5n | 4e5y | 4e79 | 4ead | 4eam | 4ear | 4eca | 4edf | 4egj |
| 4egq | 4ehi | 4ej0 | 4ekd | 4ekn | 4ekz | 4em6 | 4epi | 4eqs | 4esm |
| 4etp | 4ex4 | 4ex5 | 4exq | 4eyw | 4ez5 | 4ez8 | 4f0i | 4f0z | 4f9c |
| 4fce | 4fey | 4fg8 | 4fgw | 4fkz | 4fvy | 4fxq | 4g38 | 4g6z | 4ga6 |
| 4gac | 4gam | 4gbu | 4gcj | 4gcm | 4gdp | 4ge6 | 4gic | 4glw | 4gqs |
| 4gri | 4gut | 4gwg | 4gyp | 4h27 | 4h2g | 4h31 | 4h8a | 4hgh | 4hgv |
| 4hkt | 4hl6 | 4htg | 4htr | 4hv4 | 4hvc | 4hvs | 4i90 | 4iao | 4ic7 |
| 4ief | 4igd | 4ijn | 4ikp | 4imy | 4ip7 | 4iq8 | 4iu6 | 4iv9 | 4j15 |
| 4j1y | 4j9t | 4jb4 | 4jco | 4jnk | 4jrm | 4k7z | 4k9d | 5mdh | 7odc |
| 8cgt |  |  |  |  |  |  |  |  |  |
