# Supplementary material for: Use of an Improved Matching Algorithm to Select Scaffolds for Enzyme Design Based on a Complex Active Site Model
Source: PLoS One. 2016 May 31;11(5):e0156559. doi: 10.1371/journal.pone.0156559 (PMC4887040; doi:10.1371/journal.pone.0156559)
Supplement: S2 Table — (DOC) [file pone.0156559.s019.doc]

**S2 Table. Matching parameters for 1c2t based on complex active site model.**

| Interacting  Pair | Constraint  Type | Atom1 | Atom2 ^a^ | Atom3 ^a^ | Atom4 ^a^ | Measured  Value ^b^ | Standard  Deviation ^c^ |
| --- | --- | --- | --- | --- | --- | --- | --- |
| Asn106-NHS | Distance | ND2 | #OO26 |  |  | 2.9 | 0.1 |
|  | Angle | CG | ND2 | #OO26 |  | 132.6 | 10.0 |
|  | Angle | ND2 | #OO26 | #CO20 |  | 140.8 | 10.0 |
| Ile91-NHS | Distance | N | #OO20 |  |  | 2.9 | 0.1 |
|  | Angle | CA | N | #OO20 |  | 130.5 | 10.0 |
|  | Angle | N | #OO20 | #CO19 |  | 139.7 | 10.0 |
| Thr140-NHS | Distance | O | #NH14 |  |  | 2.7 | 0.3 |
|  | Angle | C | O | #NH14 |  | 156.8 | 30.0 |
|  | Angle | O | #NH14 | #CC28 |  | 117.1 | 30.0 |
| Asp144-NHS | Distance | OD1 | #OO26 |  |  | 2.4 | 0.3 |
|  | Angle | CG | OD1 | #OO26 |  | 121.4 | 30.0 |
|  | Angle | OD1 | #OO26 | #CO20 |  | 119.2 | 30.0 |
| His108-NHS | Distance | ND1 | #OO26 |  |  | 2.8 | 0.3 |
|  | Angle | CG | ND1 | #OO26 |  | 117.0 | 30.0 |
|  | Angle | ND1 | #OO26 | #CO20 |  | 98.1 | 30.0 |
|  | Distance | ND1 | #OO25 |  |  | 2.9 | 0.3 |
| Gly117-Asp144 | Distance | N | #OD2 |  |  | 2.9 | 0.3 |
|  | Angle | CA | N | #OD2 |  | 110.1 | 30.0 |
|  | Angle | N | #OD2 | #CG |  | 152.0 | 30.0 |
| Tyr115-His108 | Distance | O | #NE2 |  |  | 2.7 | 0.3 |
|  | Angle | C | O | #NE2 |  | 148.9 | 30.0 |
|  | Angle | O | #NE2 | #CD2 |  | 123.1 | 30.0 |

^a^: the symbol ‘#’ means the atom is on the second residue of the interacting pair.

^b^: the values are obtained from crystal structure.

^c^: the distances and angles are varied by 0.1 angstrom and 10.0 degrees for the main loop and by 0.3 angstroms and 30.0 degrees for the side loops. (The cases are the same for S2-S20 Tables)
