# Supplementary material for: Use of an Improved Matching Algorithm to Select Scaffolds for Enzyme Design Based on a Complex Active Site Model
Source: PLoS One. 2016 May 31;11(5):e0156559. doi: 10.1371/journal.pone.0156559 (PMC4887040; doi:10.1371/journal.pone.0156559)
Supplement: S3 Table — (DOC) [file pone.0156559.s020.doc]

**S3 Table. Matching parameters for 1c2t based on minimal active site model.**

| Interacting  Pair | Constraint  Type | Atom1 | Atom2 ^a^ | Atom3 ^a^ | Atom4 ^a^ | Measured  Value ^b^ | Standard  Deviation ^c^ |
| --- | --- | --- | --- | --- | --- | --- | --- |
| Asn106-NHS | Distance | ND2 | #OO26 |  |  | 2.8 | 0.1 |
|  | Angle | CG | ND2 | #OO26 |  | 132.6 | 10.0 |
|  | Angle | ND2 | #OO26 | #CO20 |  | 140.8 | 10.0 |
| Asp144-NHS | Distance | OD1 | #OO26 |  |  | 2.3 | 0.1 |
|  | Angle | CG | OD1 | #OO26 |  | 121.4 | 10.0 |
|  | Angle | OD1 | #OO26 | #CO20 |  | 119.2 | 10.0 |
| His108-NHS | Distance | ND1 | #OO26 |  |  | 2.8 | 0.3 |
|  | Angle | CG | ND1 | #OO26 |  | 117.0 | 30.0 |
|  | Angle | ND1 | #OO26 | #CO20 |  | 98.1 | 30.0 |
