# Supplementary material for: Use of an Improved Matching Algorithm to Select Scaffolds for Enzyme Design Based on a Complex Active Site Model
Source: PLoS One. 2016 May 31;11(5):e0156559. doi: 10.1371/journal.pone.0156559 (PMC4887040; doi:10.1371/journal.pone.0156559)
Supplement: S4 Table — (DOC) [file pone.0156559.s021.doc]

**S4 Table. Matching parameters for 1dqx based on complex active site model.**

| Interacting  Pair | Constraint  Type | Atom1 | Atom2 ^a^ | Atom3 ^a^ | Atom4 ^a^ | Measured  Value ^b^ | Standard  Deviation ^c^ |
| --- | --- | --- | --- | --- | --- | --- | --- |
| Asp91-BMP | Distance | OD2 | #OO20 |  |  | 2.8 | 0.1 |
|  | Angle | CG | OD2 | #OO20 |  | 119.5 | 10.0 |
|  | Angle | OD2 | #OO20 | #CN13 |  | 143.0 | 10.0 |
| Gly234-BMP | Distance | N | #OO18 |  |  | 2.8 | 0.1 |
|  | Angle | CA | N | #OO18 |  | 95.0 | 10.0 |
|  | Angle | N | #OO18 | #Ph22 |  | 125.1 | 10.0 |
| Ser154-BMP | Distance | OG | #Nh16 |  |  | 3.0 | 0.3 |
|  | Angle | CB | OG | #Nh16 |  | 120.5 | 30.0 |
|  | Angle | OG | #Nh16 | #CN11 |  | 115.0 | 30.0 |
|  | Distance | N | #ON15 |  |  | 2.8 | 0.3 |
|  | Angle | CA | N | #ON15 |  | 131.5 | 30.0 |
|  | Angle | N | #ON15 | #CN12 |  | 130.4 | 30.0 |
| Asp273-BMP | Distance | OD1 | #OH20 |  |  | 2.4 | 0.3 |
|  | Angle | CG | OD1 | #OH20 |  | 108.5 | 30.0 |
|  | Angle | OD1 | #OH20 | #CH3 |  | 136.6 | 30.0 |
|  | Distance | OD1 | #OH20 |  |  | 3.1 | 0.3 |
|  | Angle | CG | OD1 | #OH20 |  | 73.2 | 30.0 |
|  | Angle | OD1 | #OH20 | #CH3 |  | 155.3 | 30.0 |
| Lys93-BMP | Distance | NZ | #OO20 |  |  | 2.7 | 0.3 |
|  | Angle | CE | NZ | #OO20 |  | 93.8 | 30.0 |
|  | Angle | NZ | #OO20 | #CN13 |  | 121.4 | 30.0 |
| Lys93-Asp91 | Distance | NZ | #OD1 |  |  | 2.6 | 0.3 |
|  | Angle | CE | NZ | #OD1 |  | 148.7 | 30.0 |
|  | Angle | NZ | #OD1 | #CG |  | 89.0 | 30.0 |
| Lys59-Asp91 | Distance | NZ | #OD1 |  |  | 3.2 | 0.3 |
|  | Angle | CE | NZ | #OD1 |  | 88.0 | 30.0 |
|  | Angle | NZ | #OD1 | #CG |  | 111.3 | 30.0 |
| Lys59-BMP | Distance | NZ | #OH9 |  |  | 3.2 | 0.3 |
|  | Angle | CE | NZ | #OH9 |  | 106.2 | 30.0 |
|  | Angle | NZ | #OH9 | #CH2 |  | 121.2 | 30.0 |
| Ser35-Lys59 | Distance | OG | #NZ |  |  | 3.0 | 0.3 |
|  | Angle | CB | OG | #NZ |  | 127.0 | 30.0 |
|  | Angle | OG | #NZ | #CE |  | 72.6 | 30.0 |
| Thr277-BMP | Distance | OG1 | #OH20 |  |  | 2.8 | 0.3 |
|  | Angle | CB | OG1 | #OH20 |  | 110.6 | 30.0 |
|  | Angle | OG1 | #OH20 | #CH3 |  | 109.2 | 30.0 |
