# Supplementary material for: Use of an Improved Matching Algorithm to Select Scaffolds for Enzyme Design Based on a Complex Active Site Model
Source: PLoS One. 2016 May 31;11(5):e0156559. doi: 10.1371/journal.pone.0156559 (PMC4887040; doi:10.1371/journal.pone.0156559)
Supplement: S5 Table — (DOC) [file pone.0156559.s022.doc]

**S5 Table. Matching parameters for 1dqx based on minimal active site model.**

| Interacting  Pair | Constraint  Type | Atom1 | Atom2 ^a^ | Atom3 ^a^ | Atom4 ^a^ | Measured  Value ^b^ | Standard  Deviation ^c^ |
| --- | --- | --- | --- | --- | --- | --- | --- |
| Asp91-BMP | Distance | OD2 | #OO20 |  |  | 2.8 | 0.1 |
|  | Angle | CG | OD2 | #OO20 |  | 119.5 | 10.0 |
|  | Angle | OD2 | #OO20 | #CN13 |  | 143.0 | 10.0 |
| Asp273-BMP | Distance | OD1 | #OH20 |  |  | 2.4 | 0.1 |
|  | Angle | CG | OD1 | #OH20 |  | 108.5 | 10.0 |
|  | Angle | OD1 | #OH20 | #CH3 |  | 136.6 | 10.0 |
| Lys93-Asp91 | Distance | NZ | #OD1 |  |  | 2.6 | 0.3 |
|  | Angle | CE | NZ | #OD1 |  | 148.7 | 30.0 |
|  | Angle | NZ | #OD1 | #CG |  | 89.0 | 30.0 |
| Lys59-Asp91 | Distance | NZ | #OD1 |  |  | 3.2 | 0.3 |
|  | Angle | CE | NZ | #OD1 |  | 88.0 | 30.0 |
|  | Angle | NZ | #OD1 | #CG |  | 111.3 | 30.0 |
