# Supplementary material for: Use of an Improved Matching Algorithm to Select Scaffolds for Enzyme Design Based on a Complex Active Site Model
Source: PLoS One. 2016 May 31;11(5):e0156559. doi: 10.1371/journal.pone.0156559 (PMC4887040; doi:10.1371/journal.pone.0156559)
Supplement: S6 Table — (DOC) [file pone.0156559.s023.doc]

**S6 Table. Matching parameters for 1h2j based on complex active site model.**

| Interacting  Pair | Constraint  Type | Atom1 | Atom2 ^a^ | Atom3 ^a^ | Atom4 ^a^ | Measured  Value ^b^ | Standard  Deviation ^c^ |
| --- | --- | --- | --- | --- | --- | --- | --- |
| Tyr63-DCB | Distance | OH | #O38 |  |  | 2.8 | 0.1 |
|  | Angle | CZ | OH | #O38 |  | 109.7 | 10.0 |
|  | Angle | OH | #O38 | #C50 |  | 144.6 | 10.0 |
|  | Distance | OH | #O33 |  |  | 3.1 | 0.3 |
|  | Angle | CZ | OH | #O33 |  | 158.1 | 30.0 |
|  | Angle | OH | #O33 | #C47 |  | 120.5 | 30.0 |
| Ala231-DCB | Distance | O | #O35 |  |  | 2.6 | 0.1 |
|  | Angle | C | O | #O35 |  | 135.9 | 10.0 |
|  | Angle | O | #O35 | #C42 |  | 108.3 | 10.0 |
| Glu136-DCB | Distance | OE1 | #F22 |  |  | 3.1 | 0.3 |
|  | Angle | CD | OE1 | #F22 |  | 84.0 | 30.0 |
|  | Angle | OE1 | #F22 | #C51 |  | 84.6 | 30.0 |
|  | Distance | OE2 | #F22 |  |  | 3.4 | 0.3 |
|  | Angle | CD | OE2 | #F22 |  | 69.3 | 30.0 |
|  | Angle | OE2 | #F22 | #C51 |  | 105.2 | 30.0 |
|  | Distance | OE2 | #O39 |  |  | 3.1 | 0.3 |
| Glu225-DCB | Distance | OE2 | #F22 |  |  | 2.7 | 0.3 |
|  | Angle | CD | OE2 | #F22 |  | 106.5 | 30.0 |
|  | Angle | OE2 | #F22 | #C51 |  | 96.8 | 30.0 |
|  | Distance | OE1 | #F22 |  |  | 3.2 | 0.3 |
|  | Angle | CD | OE1 | #F22 |  | 96.4 | 30.0 |
|  | Angle | OE1 | #F22 | #C51 |  | 128.2 | 30.0 |
|  | Distance | OE2 | #O36 |  |  | 2.7 | 0.3 |
| Tyr199-Glu225 | Distance | OH | #OE2 |  |  | 2.6 | 0.3 |
|  | Angle | CZ | OH | #OE2 |  | 137.7 | 30.0 |
|  | Angle | OH | #OE2 | #CD |  | 121.0 | 30.0 |
| His197-Glu136 | Distance | ND1 | #OE1 |  |  | 2.6 | 0.3 |
|  | Angle | CG | ND1 | #OE1 |  | 121.4 | 30.0 |
|  | Angle | ND1 | #OE1 | #CD |  | 116.0 | 30.0 |
| Trp259-DCB | Distance | NE1 | #O34 |  |  | 3.0 | 0.3 |
|  | Angle | CE2 | NE1 | #O34 |  | 126.6 | 30.0 |
|  | Angle | NE1 | #O34 | #C46 |  | 107.8 | 30.0 |
|  | Distance | NE1 | #O37 |  |  | 3.1 | 0.3 |
|  | Angle | CD1 | NE1 | #O37 |  | 130.2 | 30.0 |
|  | Angle | NE1 | #O37 | #C47 |  | 118.5 | 30.0 |
