# Supplementary material for: Use of an Improved Matching Algorithm to Select Scaffolds for Enzyme Design Based on a Complex Active Site Model
Source: PLoS One. 2016 May 31;11(5):e0156559. doi: 10.1371/journal.pone.0156559 (PMC4887040; doi:10.1371/journal.pone.0156559)
Supplement: S7 Table — (DOC) [file pone.0156559.s024.doc]

**S7 Table. Matching parameters for 1h2j based on minimal active site model.**

| Interacting  Pair | Constraint  Type | Atom1 | Atom2 ^a^ | Atom3 ^a^ | Atom4 ^a^ | Measured  Value ^b^ | Standard  Deviation ^c^ |
| --- | --- | --- | --- | --- | --- | --- | --- |
| Glu136-DCB | Distance | OE2 | #F22 |  |  | 3.1 | 0.1 |
|  | Angle | CD | OE2 | #F22 |  | 84.0 | 10.0 |
|  | Angle | OE2 | #F22 | #C51 |  | 84.6 | 10.0 |
| Glu225-DCB | Distance | OE2 | #F22 |  |  | 2.7 | 0.1 |
|  | Angle | CD | OE2 | #F22 |  | 106.5 | 10.0 |
|  | Angle | OE2 | #F22 | #C51 |  | 96.8 | 10.0 |
