# Supplementary material for: Use of an Improved Matching Algorithm to Select Scaffolds for Enzyme Design Based on a Complex Active Site Model
Source: PLoS One. 2016 May 31;11(5):e0156559. doi: 10.1371/journal.pone.0156559 (PMC4887040; doi:10.1371/journal.pone.0156559)
Supplement: S8 Table — (DOC) [file pone.0156559.s025.doc]

**S8 Table. Matching parameters for 1jcl based on complex active site model.**

| Interacting  Pair | Constraint  Type | Atom1 | Atom2 ^a^ | Atom3 ^a^ | Atom4 ^a^ | Measured  Value ^b^ | Standard  Deviation ^c^ |
| --- | --- | --- | --- | --- | --- | --- | --- |
| Cys48-HPD | Distance | SG | #O1 |  |  | 3.2 | 0.1 |
|  | Angle | CB | SG | #O1 |  | 115.6 | 10.0 |
|  | Angle | SG | #O1 | #C1 |  | 165.6 | 10.0 |
| Thr171-HPD | Distance | O | #O4 |  |  | 2.6 | 0.1 |
|  | Angle | C | O | #O4 |  | 130.8 | 10.0 |
|  | Angle | O | #O4 | #C4 |  | 107.1 | 10.0 |
| Lys168-HPD | Distance | NZ | #C1 |  |  | 1.4 | 0.1 |
|  | Angle | CE | NZ | #C1 |  | 119.9 | 5.0 |
|  | Angle | NZ | #C1 | #C2 |  | 116.5 | 5.0 |
|  | Torsion | NZ | #C2 | #C1 | #O1 | -126.8 | 5.0 |
| Asp103-Lys168 | Distance | OD2 | #NZ |  |  | 3.1 | 0.3 |
|  | Angle | CG | OD2 | #NZ |  | 140.0 | 30.0 |
|  | Angle | OD2 | #NZ | #CE |  | 112.3 | 30.0 |
| Lys202-Asp202 | Distance | NZ | #OD2 |  |  | 2.8 | 0.3 |
|  | Angle | CE | NZ | #OD2 |  | 118.7 | 30.0 |
|  | Angle | NZ | #OD2 | #CG |  | 147.8 | 30.0 |
| Lys202-HPD | Distance | NZ | #OW |  |  | 2.8 | 0.3 |
|  | Angle | CE | NZ | #OW |  | 103.6 | 30.0 |
|  | Angle | NZ | #OW | #O1 |  | 92.9 | 30.0 |
| Ser239-HPD | Distance | OG | #O3P |  |  | 2.6 | 0.3 |
|  | Angle | CB | OG | #O3P |  | 102.9 | 30.0 |
|  | Angle | OG | #O3P | #P |  | 114.8 | 30.0 |
|  | Distance | N | #O1P |  |  | 2.9 | 0.3 |
|  | Angle | CA | N | #O1P |  | 123.9 | 30.0 |
|  | Angle | N | #O1P | #P |  | 126.7 | 30.0 |
|  | Distance | OG | #O1P |  |  | 3.3 | 0.3 |
| Asp17-Lys201 | Distance | OD2 | #NZ |  |  | 2.7 | 0.3 |
|  | Angle | CG | OD2 | #NZ |  | 121.2 | 30.0 |
|  | Angle | OD2 | #NZ | #CE |  | 105.2 | 30.0 |
| Lys138-Asp202 | Distance | NZ | #OD1 |  |  | 2.8 | 0.3 |
|  | Angle | CE | NZ | #OD1 |  | 108.3 | 30.0 |
|  | Angle | NZ | #OD1 | #CG |  | 122.6 | 30.0 |
| Lys138-Asp17 | Distance | NZ | #OD2 |  |  | 2.9 | 0.3 |
|  | Angle | CE | NZ | #OD2 |  | 98.4 | 30.0 |
|  | Angle | NZ | #OD2 | #CG |  | 148.3 | 30.0 |

^c^: angles are varied by 5.0 degrees for the interacting pair Lys168-HPD because a covalent bond is formed between the two residues (The same in S8 Table).
