# Supplementary material for: Use of an Improved Matching Algorithm to Select Scaffolds for Enzyme Design Based on a Complex Active Site Model
Source: PLoS One. 2016 May 31;11(5):e0156559. doi: 10.1371/journal.pone.0156559 (PMC4887040; doi:10.1371/journal.pone.0156559)
Supplement: S9 Table — (DOC) [file pone.0156559.s026.doc]

**S9 Table. Matching parameters for 1jcl based on minimal active site model.**

| Interacting  Pair | Constraint  Type | Atom1 | Atom2 ^a^ | Atom3 ^a^ | Atom4 ^a^ | Measured  Value ^b^ | Standard  Deviation ^c^ |
| --- | --- | --- | --- | --- | --- | --- | --- |
| Lys168-HPD | Distance | NZ | #C1 |  |  | 1.4 | 0.1 |
|  | Angle | CE | NZ | #C1 |  | 119.9 | 5.0 |
|  | Angle | NZ | #C1 | #C2 |  | 116.5 | 5.0 |
|  | Torsion | NZ | #C2 | #C1 | #O1 | -126.8 | 5.0 |
| Asp103-Lys168 | Distance | OD2 | #NZ |  |  | 3.0 | 0.1 |
|  | Angle | CG | OD2 | #NZ |  | 140.0 | 10.0 |
|  | Angle | OD2 | #NZ | #CE |  | 112.3 | 10.0 |
| Lys202-Asp202 | Distance | NZ | #OD2 |  |  | 2.8 | 0.3 |
|  | Angle | CE | NZ | #OD2 |  | 118.7 | 30.0 |
|  | Angle | NZ | #OD2 | #CG |  | 147.8 | 30.0 |
