# Supplementary material for: Use of an Improved Matching Algorithm to Select Scaffolds for Enzyme Design Based on a Complex Active Site Model
Source: PLoS One. 2016 May 31;11(5):e0156559. doi: 10.1371/journal.pone.0156559 (PMC4887040; doi:10.1371/journal.pone.0156559)
Supplement: S11 Table — (DOC) [file pone.0156559.s028.doc]

**S11 Table. Matching parameters for 1ney based on minimal active site model.**

| Interacting  Pair | Constraint  Type | Atom1 | Atom2 ^a^ | Atom3 ^a^ | Atom4 ^a^ | Measured  Value ^b^ | Standard  Deviation ^c^ |
| --- | --- | --- | --- | --- | --- | --- | --- |
| Glu163-13P | Distance | OE2 | #OH7 |  |  | 3.1 | 0.1 |
|  | Angle | CD | OE2 | #OH7 |  | 98.1 | 10.0 |
|  | Angle | OE2 | #OH7 | #CH3 |  | 74.8 | 10.0 |
| Lys11-13P | Distance | NZ | #OC9 |  |  | 3.1 | 0.1 |
|  | Angle | CE | NZ | #OC9 |  | 90.8 | 10.0 |
|  | Angle | NZ | #OC9 | #CH1 |  | 105.2 | 10.0 |
| His94-13P | Distance | NE2 | #OC9 |  |  | 2.7 | 0.3 |
|  | Angle | CD2 | NE2 | #OC9 |  | 143.1 | 30.0 |
|  | Angle | NE2 | #OC9 | #CH1 |  | 121.1 | 30.0 |
