# Supplementary material for: Use of an Improved Matching Algorithm to Select Scaffolds for Enzyme Design Based on a Complex Active Site Model
Source: PLoS One. 2016 May 31;11(5):e0156559. doi: 10.1371/journal.pone.0156559 (PMC4887040; doi:10.1371/journal.pone.0156559)
Supplement: S12 Table — (DOC) [file pone.0156559.s029.doc]

**S12 Table. Matching parameters for 1oex based on complex active site model.**

| Interacting  Pair | Constraint  Type | Atom1 | Atom2 ^a^ | Atom3 ^a^ | Atom4 ^a^ | Measured  Value ^b^ | Standard  Deviation ^c^ |
| --- | --- | --- | --- | --- | --- | --- | --- |
| Asp217-LOV | Distance | OD1 | #O12 |  |  | 2.6 | 0.1 |
|  | Angle | CG | OD1 | #O12 |  | 104.3 | 10.0 |
|  | Angle | OD1 | #O12 | #CH3 |  | 120.8 | 10.0 |
|  | Distance | OD2 | #O12 |  |  | 3.0 | 0.3 |
|  | Angle | CG | OD2 | #O12 |  | 85.6 | 30.0 |
|  | Angle | OD2 | #O12 | #CH3 |  | 163.3 | 30.0 |
| Gly219-LOV | Distance | O | #N14 |  |  | 3.0 | 0.1 |
|  | Angle | C | O | #N14 |  | 123.3 | 10.0 |
|  | Angle | O | #N14 | #CH2 |  | 104.4 | 10.0 |
| Asp35-LOV | Distance | OD1 | #O12 |  |  | 2.6 | 0.3 |
|  | Angle | CG | OD1 | #O12 |  | 112.4 | 30.0 |
|  | Angle | OD1 | #O12 | #CH3 |  | 129.0 | 30.0 |
|  | Distance | OD2 | #O12 |  |  | 3.3 | 0.3 |
|  | Angle | CG | OD2 | #O12 |  | 78.4 | 30.0 |
|  | Angle | OD2 | #O12 | #CH3 |  | 97.2 | 30.0 |
| Ser38-Asp35 | Distance | OG | #OD2 |  |  | 2.6 | 0.3 |
|  | Angle | CB | OG | #OD2 |  | 105.6 | 30.0 |
|  | Angle | OG | #OD2 | #CG |  | 122.6 | 30.0 |
| Thr220-Asp217 | Distance | OG1 | #OD1 |  |  | 2.8 | 0.3 |
|  | Angle | CB | OG1 | #OD1 |  | 117.5 | 30.0 |
|  | Angle | OG1 | #OD1 | #CG |  | 116.3 | 30.0 |
| Gly78-LOV | Distance | N | #O15 |  |  | 2.8 | 0.3 |
|  | Angle | CA | N | #O15 |  | 126.4 | 30.0 |
|  | Angle | N | #O15 | #C13 |  | 160.1 | 30.0 |
