# Supplementary material for: Use of an Improved Matching Algorithm to Select Scaffolds for Enzyme Design Based on a Complex Active Site Model
Source: PLoS One. 2016 May 31;11(5):e0156559. doi: 10.1371/journal.pone.0156559 (PMC4887040; doi:10.1371/journal.pone.0156559)
Supplement: S13 Table — (DOC) [file pone.0156559.s030.doc]

**S13 Table. Matching parameters for 1oex based on minimal active site model.**

| Interacting  Pair | Constraint  Type | Atom1 | Atom2 ^a^ | Atom3 ^a^ | Atom4 ^a^ | Measured  Value ^b^ | Standard  Deviation ^c^ |
| --- | --- | --- | --- | --- | --- | --- | --- |
| Asp35-LOV | Distance | OD1 | #O12 |  |  | 2.6 | 0.1 |
|  | Angle | CG | OD1 | #O12 |  | 112.4 | 10.0 |
|  | Angle | OD1 | #O12 | #CH3 |  | 129.0 | 10.0 |
| Asp217-LOV | Distance | OD1 | #O12 |  |  | 2.6 | 0.1 |
|  | Angle | CG | OD1 | #O12 |  | 104.3 | 10.0 |
|  | Angle | OD1 | #O12 | #CH3 |  | 120.8 | 10.0 |
