# Supplementary material for: Use of an Improved Matching Algorithm to Select Scaffolds for Enzyme Design Based on a Complex Active Site Model
Source: PLoS One. 2016 May 31;11(5):e0156559. doi: 10.1371/journal.pone.0156559 (PMC4887040; doi:10.1371/journal.pone.0156559)
Supplement: S14 Table — (DOC) [file pone.0156559.s031.doc]

**S14 Table. Matching parameters for 1p6o based on complex active site model.**

| Interacting  Pair | Constraint  Type | Atom1 | Atom2 ^a^ | Atom3 ^a^ | Atom4 ^a^ | Measured  Value ^b^ | Standard  Deviation ^c^ |
| --- | --- | --- | --- | --- | --- | --- | --- |
| Cys89-HPY | Distance | SG | #Zn1 |  |  | 2.3 | 0.1 |
|  | Angle | CB | SG | #Zn1 |  | 112.5 | 10.0 |
|  | Angle | SG | #Zn1 | #OH5 |  | 108.2 | 10.0 |
|  | Distance | N | #OH5 |  |  | 3.0 | 0.3 |
| Cys92-HPY | Distance | SG | #Zn1 |  |  | 2.3 | 0.1 |
|  | Angle | CB | SG | #Zn1 |  | 103.7 | 10.0 |
|  | Angle | SG | #Zn1 | #OH5 |  | 102.9 | 10.0 |
| Glu62-HPY | Distance | OE1 | #NH4 |  |  | 2.8 | 0.3 |
|  | Angle | CD | OE1 | #NH4 |  | 117.0 | 30.0 |
|  | Angle | OE1 | #NH4 | #CH2 |  | 104.0 | 30.0 |
|  | Distance | OE2 | #NH5 |  |  | 2.5 | 0.3 |
| His60-HPY | Distance | ND1 | #Zn1 |  |  | 2.0 | 0.3 |
|  | Angle | CE1 | ND1 | #Zn1 |  | 123.6 | 30.0 |
|  | Angle | ND1 | #Zn1 | #OH5 |  | 113.4 | 30.0 |
| Asn49-HPY | Distance | ND2 | #ON5 |  |  | 2.9 | 0.3 |
|  | Angle | CG | ND2 | #ON5 |  | 121.7 | 30.0 |
|  | Angle | ND2 | #ON5 | #CH7 |  | 123.5 | 30.0 |
| Asp153-HPY | Distance | OD2 | #NH3 |  |  | 2.7 | 0.3 |
|  | Angle | CG | OD2 | #NH3 |  | 140.6 | 30.0 |
|  | Angle | OD2 | #NH3 | #CH9 |  | 106.4 | 30.0 |
| Asp153-Asn49 | Distance | OD1 | #ND2 |  |  | 2.9 | 0.3 |
|  | Angle | CG | OD1 | #ND2 |  | 98.8 | 30.0 |
|  | Angle | OD1 | #ND2 | #CG |  | 126.2 | 30.0 |
|  | Distance | OD2 | #ND2 |  |  | 3.1 | 0.3 |
|  | Angle | CG | OD2 | #ND2 |  | 88.1 | 30.0 |
|  | Angle | OD2 | #ND2 | #CG |  | 141.8 | 30.0 |
