# Supplementary material for: Use of an Improved Matching Algorithm to Select Scaffolds for Enzyme Design Based on a Complex Active Site Model
Source: PLoS One. 2016 May 31;11(5):e0156559. doi: 10.1371/journal.pone.0156559 (PMC4887040; doi:10.1371/journal.pone.0156559)
Supplement: S15 Table — (DOC) [file pone.0156559.s032.doc]

**S15 Table. Matching parameters for 1p6o based on minimal active site model.**

| Interacting  Pair | Constraint  Type | Atom1 | Atom2 ^a^ | Atom3 ^a^ | Atom4 ^a^ | Measured  Value ^b^ | Standard  Deviation ^c^ |
| --- | --- | --- | --- | --- | --- | --- | --- |
| Cys89-HPY | Distance | SG | #Zn1 |  |  | 2.3 | 0.1 |
|  | Angle | CB | SG | #Zn1 |  | 112.5 | 10.0 |
|  | Angle | SG | #Zn1 | #OH5 |  | 108.2 | 10.0 |
| Glu62-HPY | Distance | OE1 | #NH4 |  |  | 2.8 | 0.1 |
|  | Angle | CD | OE1 | #NH4 |  | 117.0 | 10.0 |
|  | Angle | OE1 | #NH4 | #CH2 |  | 104.0 | 10.0 |
| Cys92-HPY | Distance | SG | #Zn1 |  |  | 2.3 | 0.3 |
|  | Angle | CB | SG | #Zn1 |  | 103.7 | 30.0 |
|  | Angle | SG | #Zn1 | #OH5 |  | 102.9 | 30.0 |
| His60-HPY | Distance | ND1 | #Zn1 |  |  | 2.0 | 0.3 |
|  | Angle | CE1 | ND1 | #Zn1 |  | 123.6 | 30.0 |
|  | Angle | ND1 | #Zn1 | #OH5 |  | 113.4 | 30.0 |
