# Supplementary material for: Use of an Improved Matching Algorithm to Select Scaffolds for Enzyme Design Based on a Complex Active Site Model
Source: PLoS One. 2016 May 31;11(5):e0156559. doi: 10.1371/journal.pone.0156559 (PMC4887040; doi:10.1371/journal.pone.0156559)
Supplement: S16 Table — (DOC) [file pone.0156559.s033.doc]

**S16 Table. Matching parameters for 3vgc based on complex active site model.**

| Interacting  Pair | Constraint  Type | Atom1 | Atom2 ^a^ | Atom3 ^a^ | Atom4 ^a^ | Measured  Value ^b^ | Standard  Deviation ^c^ |
| --- | --- | --- | --- | --- | --- | --- | --- |
| Ser175-SRB | Distance | OG | #B |  |  | 1.5 | 0.1 |
|  | Angle | CB | OG | #B |  | 112.2 | 5.0 |
|  | Angle | OG | #B | #OH5 |  | 108.2 | 5.0 |
|  | Torsion | OG | #OH5 | #B | #OH6 | 121.3 | 5.0 |
|  | Distance | N | #OH6 |  |  | 3.0 | 0.3 |
|  | Angle | CA | N | #OH6 |  | 95.3 | 30.0 |
|  | Angle | N | #OH6 | #B |  | 100.8 | 30.0 |
| Gly173-SRB | Distance | N | #OH6 |  |  | 2.9 | 0.1 |
|  | Angle | CA | N | #OH6 |  | 117.5 | 10.0 |
|  | Angle | N | #OH6 | #B |  | 163.4 | 10.0 |
| His42-SRB | Distance | NE2 | #OH5 |  |  | 2.7 | 0.3 |
|  | Angle | CD2 | NE2 | #OH5 |  | 106.0 | 30.0 |
|  | Angle | NE2 | #OH5 | #B |  | 93.4 | 30.0 |
|  | Distance | NE2 | #NH4 |  |  | 2.9 | 0.3 |
|  | Angle | CE1 | NE2 | #NH4 |  | 104.3 | 30.0 |
|  | Angle | NE2 | #NH4 | #CH1 |  | 110.4 | 30.0 |
| His42-Ser175 | Distance | NE2 | #OG |  |  | 2.7 | 0.3 |
|  | Angle | CE1 | ND1 | #OG |  | 91.8 | 30.0 |
|  | Angle | NE2 | #OG | #CB |  | 99.1 | 30.0 |
| Asp87-His42 | Distance | OD2 | #ND1 |  |  | 2.7 | 0.3 |
|  | Angle | CG | OD2 | #ND1 |  | 121.8 | 30.0 |
|  | Angle | OD2 | #ND1 | #CG |  | 132.8 | 30.0 |
|  | Distance | OD1 | #N |  |  | 2.8 | 0.3 |
|  | Angle | CG | OD1 | #N |  | 146.0 | 30.0 |
|  | Angle | OD1 | #N | #CA |  | 126.5 | 30.0 |
| Ser194-Asp87 | Distance | OG | #OD2 |  |  | 2.8 | 0.3 |
|  | Angle | CB | OG | #OD2 |  | 108.6 | 30.0 |
|  | Angle | OG | #OD2 | #CG |  | 124.2 | 30.0 |
| Ser194-SRB | Distance | O | #NH4 |  |  | 3.0 | 0.3 |
|  | Angle | C | O | #NH4 |  | 147.4 | 30.0 |
|  | Angle | O | #NH4 | #CH1 |  | 121.8 | 30.0 |

^c^: angles are varied by 5.0 degrees for the interacting pair Ser175-SRB because a covalent bond is formed between the two residues (The same in S16 Table).
