# Supplementary material for: Use of an Improved Matching Algorithm to Select Scaffolds for Enzyme Design Based on a Complex Active Site Model
Source: PLoS One. 2016 May 31;11(5):e0156559. doi: 10.1371/journal.pone.0156559 (PMC4887040; doi:10.1371/journal.pone.0156559)
Supplement: S17 Table — (DOC) [file pone.0156559.s034.doc]

**S17 Table. Matching parameters for 3vgc based on minimal active site model.**

| Interacting  Pair | Constraint  Type | Atom1 | Atom2 ^a^ | Atom3 ^a^ | Atom4 ^a^ | Measured  Value ^b^ | Standard  Deviation ^c^ |
| --- | --- | --- | --- | --- | --- | --- | --- |
| Ser175-SRB | Distance | OG | #B |  |  | 1.5 | 0.1 |
|  | Angle | CB | OG | #B |  | 112.2 | 5.0 |
|  | Angle | OG | #B | #OH5 |  | 108.2 | 5.0 |
|  | Torsion | OG | #OH5 | #B | #OH6 | 121.3 | 5.0 |
| His42-SRB | Distance | NE2 | #OH5 |  |  | 2.7 | 0.1 |
|  | Angle | CD2 | NE2 | #OH5 |  | 106.0 | 10.0 |
|  | Angle | NE2 | #OH5 | #B |  | 93.4 | 10.0 |
| Asp87-His42 | Distance | OD2 | #ND1 |  |  | 2.7 | 0.3 |
|  | Angle | CG | OD2 | #ND1 |  | 121.8 | 30.0 |
|  | Angle | OD2 | #ND1 | #CG |  | 132.8 | 30.0 |
