# Supplementary material for: Use of an Improved Matching Algorithm to Select Scaffolds for Enzyme Design Based on a Complex Active Site Model
Source: PLoS One. 2016 May 31;11(5):e0156559. doi: 10.1371/journal.pone.0156559 (PMC4887040; doi:10.1371/journal.pone.0156559)
Supplement: S18 Table — (DOC) [file pone.0156559.s035.doc]

**S18 Table. Matching parameters for 4fua based on complex active site model.**

| Interacting  Pair | Constraint  Type | Atom1 | Atom2 ^a^ | Atom3 ^a^ | Atom4 ^a^ | Measured  Value ^b^ | Standard  Deviation ^c^ |
| --- | --- | --- | --- | --- | --- | --- | --- |
| His155-PGH | Distance | NE2 | #Zn1 |  |  | 2.0 | 0.1 |
|  | Angle | CD2 | NE2 | #Zn1 |  | 130.4 | 10.0 |
|  | Angle | NE2 | #Zn1 | #OO6 |  | 98.1 | 10.0 |
| Thr43-PGH | Distance | OG1 | #OO8 |  |  | 2.9 | 0.1 |
|  | Angle | CB | OG1 | #OO8 |  | 96.0 | 10.0 |
|  | Angle | OG1 | #OO8 | #Ph11 |  | 122.0 | 10.0 |
| His94-PGH | Distance | NE2 | #Zn1 |  |  | 2.0 | 0.3 |
|  | Angle | CD2 | NE2 | #Zn1 |  | 127.3 | 10.0 |
|  | Angle | NE2 | #Zn1 | #OO5 |  | 90.4 | 10.0 |
| His92-PGH | Distance | NE2 | #Zn1 |  |  | 2.1 | 0.3 |
|  | Angle | CD2 | NE2 | #Zn1 |  | 124.5 | 10.0 |
|  | Angle | NE2 | #Zn1 | #OO5 |  | 97.0 | 10.0 |
| Gly44-PGH | Distance | N | #OO9 |  |  | 2.9 | 0.3 |
|  | Angle | CA | N | #OO9 |  | 114.4 | 30.0 |
|  | Angle | N | #OO9 | #Ph11 |  | 130.6 | 30.0 |
