# Supplementary material for: Use of an Improved Matching Algorithm to Select Scaffolds for Enzyme Design Based on a Complex Active Site Model
Source: PLoS One. 2016 May 31;11(5):e0156559. doi: 10.1371/journal.pone.0156559 (PMC4887040; doi:10.1371/journal.pone.0156559)
Supplement: S21 Table — (DOC) [file pone.0156559.s038.doc]

**S21 Table. Matching parameters for 6cpa based on minimal active site model.**

| Interacting  Pair | Constraint  Type | Atom1 | Atom2 ^a^ | Atom3 ^a^ | Atom4 ^a^ | Measured  Value ^b^ | Standard  Deviation ^c^ |
| --- | --- | --- | --- | --- | --- | --- | --- |
| His69-ZAF | Distance | ND1 | #Zn1 |  |  | 2.1 | 0.1 |
|  | Angle | CE1 | ND1 | #Zn1 |  | 144.9 | 10.0 |
|  | Angle | ND1 | #Zn1 | #O19 |  | 91.8 | 10.0 |
| Glu270-ZAF | Distance | OE2 | #O20 |  |  | 2.2 | 0.1 |
|  | Angle | CD | OE2 | #O20 |  | 123.4 | 10.0 |
|  | Angle | OE2 | #O20 | #P22 |  | 136.8 | 10.0 |
| His196-ZAF | Distance | ND1 | #Zn1 |  |  | 2.1 | 0.3 |
|  | Angle | CE1 | ND1 | #Zn1 |  | 120.4 | 30.0 |
|  | Angle | ND1 | #Zn1 | #O19 |  | 110.0 | 30.0 |
| Glu72-ZAF | Distance | OE2 | #Zn1 |  |  | 2.3 | 0.3 |
|  | Angle | CD | OE2 | #Zn1 |  | 91.4 | 30.0 |
|  | Angle | OE2 | #Zn1 | #O19 |  | 136.0 | 30.0 |
