# Supplementary material for: Use of an Improved Matching Algorithm to Select Scaffolds for Enzyme Design Based on a Complex Active Site Model
Source: PLoS One. 2016 May 31;11(5):e0156559. doi: 10.1371/journal.pone.0156559 (PMC4887040; doi:10.1371/journal.pone.0156559)
Supplement: S22 Table — (DOC) [file pone.0156559.s039.doc]

**S22 Table. RMS deviations of residues in native matches and CPU running time of ProdaMatch for ten scaffolds.**

| PDB | RMSD of residues ^a^ | | | | | | | | Time (hr) ^b^ |
| --- | --- | --- | --- | --- | --- | --- | --- | --- | --- |
| 1c2t | Asn106 | His108 | Asp144 | Thr140 | Ile91 | Gly117 | Tyr115 |  |  |
|  | 0.20 (2.15) | 0.24 (0.46) | 0.18 (0.59) | 0.03 | 0.04 | 0.02 | 0.04 |  | 19.0 (20.8) |
| 1dqx | Asp91 | Asp273 | Lys93 | Lys59 | Ser154 | Gly234 | Ser35 | Thr277 |  |
|  | 1.38 (0.70) | 0.32 (1.58) | 0.91 (2.10) | 0.84 (2.47) | 0.80 | 0.04 | 1.18 | 0.74 | 6.0 (21.0) |
| 1h2j | Glu225 | Glu136 | Tyr63 | Ala231 | Tyr199 | His197 | Trp259 |  |  |
|  | 0.53 (2.45) | 1.33 (1.58) | 1.85 | 0.04 | 0.99 | 1.03 | 0.72 |  | 9.4 (9.8) |
| 1jcl | Lys168 | Asp103 | Lys202 | Cys48 | Thr171 | Ser239 | Asp17 | Lys138 |  |
|  | 0.59 (0.86) | 0.37 (1.42) | 0.57 (1.52) | 0.82 | 0.04 | 0.06 | 0.26 | 1.01 | 4.6 (24.3) |
| 1ney | Glu164 | His94 | Lys11 | Gly231 | Gly170 | Glu96 |  |  |  |
|  | 1.38 (1.46) | 0.48 (0.91) | 0.44 (0.90) | 0.04 | 0.04 | 0.79 |  |  | 4.0 (4.0) |
| 1oex | Asp35 | Asp217 | Gly219 | Gly78 | Ser38 | Thr220 |  |  |  |
|  | 0.06 (0.65) | 0.71 (0.97) | 0.04 | 0.03 | 0.16 | 0.31 |  |  | 4.2 (0.4) |
| 1p6o | Cys89 | Cys92 | His60 | Glu62 | Asn49 | Asp153 |  |  |  |
|  | 0.06 (0.23) | 0.05 (0.79) | 0.56 (0.90) | 0.19 (0.66) | 0.84 | 0.65 |  |  | 0.9 (1.2) |
| 3vgc | Ser175 | His42 | Asp87 | Gly173 | Ser194 |  |  |  |  |
|  | 0.09 (0.15) | 0.42 (0.54) | 0.17 (1.05) | 0.04 | 0.10 |  |  |  | 0.3 (4.0) |
| 4fua | His155 | His94 | His92 | Thr43 | Gly44 |  |  |  |  |
|  | 0.16 (0.44) | 0.40 (0.42) | 0.32 (1.88) | 0.15 | 0.04 |  |  |  | 7.2 (3.9) |
| 6cpa | His69 | Glu270 | His196 | Glu72 | Asp142 | Arg127 |  |  |  |
|  | 0.39 (1.13) | 1.73 (1.49) | 1.07 (1.28) | 1.04 (1.00) | 0.53 | 0.74 |  |  | 60.3 (24.1) |

^a^: The numbers outside and inside the parentheses are the RMS deviations of the residues in the calculated complex and minimal active site, respectively.

^b^: The numbers outside and inside the parentheses are the computation time of the matching process using the complex and minimal active site model, respectively.
