# Supplementary material for: Use of an Improved Matching Algorithm to Select Scaffolds for Enzyme Design Based on a Complex Active Site Model
Source: PLoS One. 2016 May 31;11(5):e0156559. doi: 10.1371/journal.pone.0156559 (PMC4887040; doi:10.1371/journal.pone.0156559)
Supplement: S23 Table — (DOC) [file pone.0156559.s040.doc]

**S23 Table. Hydrogen bonding recapitulation results for seven scaffolds based on different active site models.**

| PDB | Hydrogen bonds ^a^ | | |
| --- | --- | --- | --- |
| 1c2t | (N106)ND2-OO26(NHS) | (N106)ND2-NE2(H136) | (H108)ND1-OO25(NHS)/M |
|  | (H108)NE2-O(Y115) | (D144)OD1-OO26(NHS) | (D144)N-ON16(NHS)/M |
|  | (D144)OD1-N(G117) | (I91)N-OO22(NHS)/M | (T140)O-NH14(NHS)/M |
|  | (L92)O-NH13(NHS)/M | (L92)N-NH12(NHS)/M | (R64)NH1-OO23(NHS)/M |
|  | (R64)NH2-OO22(NHS)/M |  |  |
| 1dqx | (D91)OD2-OO20(BMP) | (D91)OD2-NZ(K59) | (D91)OD1-NZ(K93)/M |
|  | (D91)OD2-NZ(K93)/M | (D273)OD1-OH20(BMP)/M | (D273)OD2-NZ(K93)/C |
|  | (K93)NZ-OO20(BMP)/M | (K59)NZ-OH9(BMP)/M | (K59)NZ-OG(S35)/MC |
|  | (S154)OG-Nh16(BMP)/M | (S154)N-ON15(BMP)/M | (R235)NH1-OO19(BMP)/M |
|  | (R235)NH1-OO17(BMP)/M | (R235)N-OO19(BMP)/M | (D37)OD2-OH9(BMP)/M |
|  | (Y217)OH-OO17(BMP)/M | (G234)N-OO18(BMP)/M | (Q215)NE2-OO17(BMP)/M |
|  | (Q215)NE2-ON14(BMP)/M |  |  |
| 1h2j | (E225)OE2-OH(Y199) | (E255)OE1-NH1(R59)/M | (E136)OE2-ND1(H197) |
|  | (Y63)OH-O38(DCB)/MC | (Y63)OH-O33(DCB)/MC | (A231)O-O35(DCB)/M |
|  | (R59)NH1-OG(S30)/M | (R59)NH2-OE2(E132)/M | (R59)NE-OE1(E132)/M |
|  | (H98)NE2-O38(DCB)/M | (N135)ND2-F22(DCB) | (E266)OE2-O34(DCB)/M |
|  | (W259)NE1-O34(DCB)/MC |  |  |
| 1jcl | (K168)NZ-OD2(D103)/M | (D103)OD2-NZ(K202) | (D103)OD1-NZ(K138) |
|  | (K202)NZ-OW(HPD)/M | (C48)SG-O1(HPD) | (T171)O-O4(HPD)/M |
|  | (S239)OG-O3P(HPD)/M | (S239)N-O1P(HPD)/M | (D17)OD2-NZ(K138)/M |
|  | (G206)N-O2P(HPD)/M | (T19)OG1-OW(HPD) |  |
| 1ney | (E164)OE1-OH7(13P)/MC | (E164)OE2-OH7(13P)/MC | (H94)NE2-OH7(13P)/C |
|  | (H94)NE2-OC9(13P) | (K11)NZ-OC9(13P) | (K11)NZ-OH6(13P)/M |
|  | (G231)N-OH5(13P)/M | (G170)N-OC8(13P) | (E96)OE1-NZ(K11) |
|  | (E96)N-ND1(H94) | (SER210)N-OC8(13P)/M | (G232)N-OH4(13P)/M |
|  | (N9)ND2-OH7(13P) | (N9)OD1-N(K11) |  |
| 1oex | (D35)OD1-O12(LOV) | (D35)OD2-O12(LOV)/M | (D35)OD1-N(G37) |
|  | (D35)OD2-OG(S38) | (D217)OD1-O12(LOV) | (D217)OD1-OG1(T220) |
|  | (D217)OD2-N(G219) | (G78)N-O15(LOV)/M | (G219)O-N14(LOV)/M |
| 3vgc | (S175)OG-NE2(H42) | (S175)N-OH6(SRB)/M | (H42)NE2-OH5(SRB) |
|  | (H42)NE2-NH4(SRB) | (H42)ND1-OD2(D87) | (H42)N-OD1(D87) |
|  | (G173)N-OH6(SRB) | (S194)OG-OD2(D87) | (S194)O-NH4(SRB) |

^a^: the alphabet ‘M’ and/or ‘C’ after ‘/’ mean that the hydrogen bond is **not** recapitulated during the repacking calculations with the TS position obtained from the native match of **minimal** active site model and/or the native match of **complex** active site model.
