# Supplementary material for: Use of an Improved Matching Algorithm to Select Scaffolds for Enzyme Design Based on a Complex Active Site Model
Source: PLoS One. 2016 May 31;11(5):e0156559. doi: 10.1371/journal.pone.0156559 (PMC4887040; doi:10.1371/journal.pone.0156559)
Supplement: S25 Table — (DOC) [file pone.0156559.s042.doc]

**S25** Table. Matching parameters for cephalexin based on classical catalytic triad motif.

| Interacting  Pair | Constraint  Type | Atom1 | Atom2^a^ | Atom3^a^ | Atom4^a^ | Middle  Value | Standard  Deviation |
| --- | --- | --- | --- | --- | --- | --- | --- |
| SER1-TS | Distance | OG | #C15 |  |  | 1.5 | 0.2 |
|  | Angle | CB | OG | #C15 |  | 110.0 | 10.0 |
|  | Angle | OG | #C8 | #O16 |  | 110.0 | 10.0 |
| SER1-HIS2 | Distance | OG | #NE2 |  |  | 2.8 | 0.4 |
|  | Angle | CB | OG | #NE2 |  | 110.0 | 40.0 |
|  | Angle | OG | #NE2 | #CD2 |  | 120.0 | 40.0 |
| HSD2-ASP3 | Distance | ND1 | #OD1 |  |  | 2.8 | 0.4 |
|  | Angle | CG | ND1 | #OD1 |  | 120.0 | 40.0 |
|  | Angle | ND1 | #OD1 | #CG |  | 120.0 | 40.0 |
|  | Angle | ND1 | HD1 | #OD1 |  | 150.0 | 30.0 |
| ASP3-ALA6 | Distance | OD2 | #N |  |  | 2.8 | 0.4 |
|  | Angle | CG | OD2 | #N |  | 120.0 | 40.0 |
|  | Angle | OD2 | #N | #CA |  | 120.0 | 40.0 |
|  | Angle | OD2 | #HN | #N |  | 150.0 | 30.0 |
| ALA4-TS | Distance | N | #O16 |  |  | 2.8 | 0.4 |
|  | Angle | N | #O16 | #C15 |  | 110.0 | 40.0 |
|  | Angle | CA | N | #O16 |  | 120.0 | 40.0 |
|  | Angle | N | HN | #O16 |  | 150.0 | 30.0 |
| ALA5-TS | Distance | N | #O16 |  |  | 2.8 | 0.4 |
|  | Angle | N | #O16 | #C15 |  | 110.0 | 40.0 |
|  | Angle | CA | N | #O16 |  | 120.0 | 40.0 |
|  | Angle | N | HN | #O16 |  | 150.0 | 30.0 |

^a^: the atoms of the second residue are prefixed with ‘#’.
